# Oxidative Stress and Hormone-Regulated Dermal Papilla Cell-Targeted Nanomodulators: Reverse Cellular Senescence for Androgenetic Alopecia Therapy

**DOI:** 10.34133/bmr.0333

**Published:** 2026-03-11

**Authors:** Lan Lan, Qingde Zhou, Shuangxue Pan, Hui Liu, Yongzhong Du, Cuiping Guan, Xiuzu Song, Wei Wang

**Affiliations:** ^1^ Department of Dermatology, Affiliated Hangzhou Dermatology Hospital, Zhejiang University School of Medicine, Hangzhou Third People’s Hospital, Hangzhou 310009, China.; ^2^ Department of Pharmacy, Affiliated Hangzhou Dermatology Hospital, Zhejiang University School of Medicine, Hangzhou Third People’s Hospital, Hangzhou 310009, China.; ^3^ School of Pharmacy, Hangzhou Normal University, Hangzhou, Zhejiang 311121, China.; ^4^ Hangzhou Third Hospital Affiliated Zhejiang Chinese Medical University, Hangzhou 310009, China.; ^5^ State Key Laboratory of Advanced Drug Delivery and Release Systems, Institute of Pharmaceutics, College of Pharmaceutical Sciences, Zhejiang University, Hangzhou 310058, China.

## Abstract

Androgenetic alopecia (AGA), a prevalent form of hair loss disorder, is pathologically characterized by dermal papilla cell (DPC) senescence driven by the dual pathogenic effects of excessive dihydrotestosterone accumulation and reactive-oxygen-species-mediated oxidative stress. Current clinical treatment strategies are challenged by poor hair follicle targeting, short retention times, and limited efficacy due to single-pathway interventions. To address these limitations, we developed a DPC-targeted, dual-functional finasteride/cerium oxide nanoparticle (L-LP-Fi/CeNP) drug delivery system designed to synergistically counteract DPC senescence through concurrent dihydrotestosterone inhibition and reactive oxygen species scavenging. By actively targeting the DPC-specific surface marker leptin receptor, this system notable enhanced cutaneous penetration depth and prolonged intrafollicular drug retention. Within the pathological microenvironment, the combined action of finasteride and CeNPs down-regulated senescence markers (p16/pRb) via dual-pathway synergy, effectively reversing cellular senescence and restoring the hair-inductive capacity of DPCs. In AGA mouse models, L-LP-Fi/CeNPs exhibited hair regenerative efficacy comparable to, and in some aspects modestly improved over, that of minoxidil, the current clinical standard treatment. This study presents a novel targeted therapeutic strategy combining small-molecule drug synergism with nanotechnology, which offers a promising prospect for AGA treatment.

## Introduction

Androgenetic alopecia (AGA) is a prevalent hair loss disorder affecting 80% of males and 40% of females globally, adversely impacting their psychoemotional health and quality of life [1]. The etiology of AGA is primarily associated with dihydrotestosterone (DHT), a potent derivative of testosterone (T), which induces the miniaturization of hair follicles (HFs) and causes the transformation of terminal hair into vellus hair [2]. Current Food and Drug Administration-approved treatments for AGA, including topical minoxidil and oral finasteride, demonstrate limited efficacy due to individual variability, the requirement for prolonged use, and tolerability-related concerns [3,4]. Consequently, exploring the underlying mechanisms of AGA and developing targeted topical delivery systems have emerged as promising strategies to enhance treatment adherence and improve therapeutic performance.

Dermal papilla cells (DPCs), a crucial mesenchymal component located at the base of HFs, play a vital role in regulating hair formation and promoting hair regeneration through reciprocal epithelial–mesenchymal interactions [5–8]. Dysfunction linked to the premature senescence of DPCs is a potential mechanism underlying AGA [9,10]. In AGA, DPCs experience early aging in response to elevated DHT levels and oxidative stress, which impairs their capacity to activate epithelial hair follicle stem cells (HFSCs) and disrupts the normal HF cycle [11–14]. DHT binds to the androgen receptor (AR) on DPCs, forming the AR–DHT complex that imitates the aging process and up-regulates the expression of hair growth inhibitory factors, such as transforming growth factor β (TGF-β), thereby promoting HF regression [10]. Concurrently, oxidative stress resulting from excessive reactive oxygen species (ROS) in the perifollicular microenvironment overwhelms intrinsic antioxidant defense mechanisms, leading to oxidative damage that accelerates cellular senescence and inhibits the transition from the telogen to the anagen phase [9]. Therefore, strategies aimed at targeting DPCs and inhibiting DHT- and ROS-induced senescence in DPCs are critical for the effective treatment of AGA.

Given the reprogramming and HF-inducing capacities of DPCs, various strategies have targeted DPCs for therapeutic purposes. These strategies include leveraging the homing effect of mesenchymal-stem-cell-derived exosomes and the chemotactic properties of platelet-rich plasma therapy [15,16]. However, these approaches often suffer from imprecise targeting and encounter substantial challenges related to production and purification. Recent studies, including our own, have identified the leptin receptor (LEPR) as a signature gene for DPCs, with a stable and specific expression pattern throughout the HF cycle in DPCs, suggesting that LEPR is a promising target for DPC-based therapies.

Finasteride, a 5α-reductase inhibitor, reduces DHT levels by blocking the conversion of T to DHT [17]. Topical administration of finasteride has been reported to limit systemic exposure compared with oral administration while maintaining therapeutic activity at the scalp. Additionally, cerium oxide nanoparticles (CeNPs) exhibit ROS-scavenging activity in a recyclable manner by reversibly binding to oxygen atoms and shuttling between the Ce^3+^ and Ce^4+^ states on their surface [18]. The Ce^3+^ sites effectively remove ROS via superoxide dismutase (SOD) mimetic activity and neutralize hydroxyl radicals (·OH) through redox reactions, whereas the Ce^4+^ sites facilitate the oxidation of hydrogen peroxide (H_2_O_2_) via catalase (CAT) mimetic activity [19,20]. Given their efficacy in addressing ROS-related diseases such as psoriasis and acute kidney injury, CeNPs have the potential to reduce ROS levels in AGA [21,22]. However, the clinical application of both finasteride and CeNPs is constrained by poor water solubility, low transdermal absorption, and a lack of targeted delivery capabilities [23,24].

**Fig. 1.**
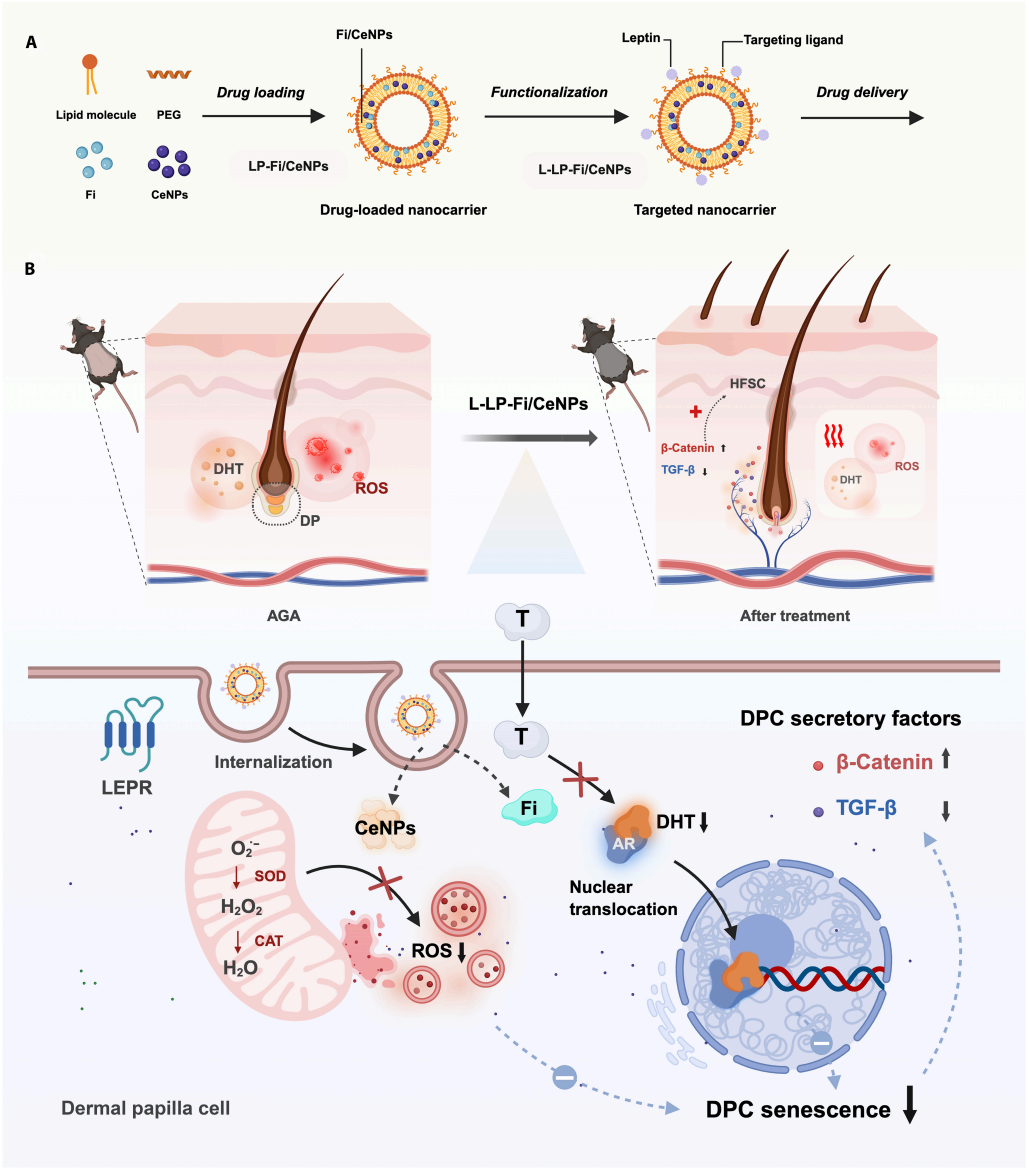
Schematic illustration of the fabrication process (A) and therapeutic AGA mechanism (B) of DPC-targeted nanocarriers (L-LP-Fi/CeNPs) that synergistically inhibit DPC senescence. DPC, dermal papilla cell; PEG, polyethylene glycol; CeNP, cerium oxide nanoparticle; Fi, finasteride; L-LP, leptin-functionalized, co-loaded liposome; DP, dermal papilla; DHT, dihydrotestosterone; ROS, reactive oxygen species; HFSC, hair follicle stem cell; TGF-β, transforming growth factor β; AGA, androgenetic alopecia; LEPR, leptin receptor; T, testosterone; SOD, superoxide dismutase; CAT, catalase.

To address these challenges, we developed a leptin-functionalized liposomal nanomodulator (L-LP-Fi/CeNPs) that integrates finasteride and CeNPs within a targeted delivery platform designed for DPCs [25–27]. This liposomal system provides enhanced solubility and delivery efficiency for finasteride and CeNPs and facilitates localized drug retention within the skin [28,29]. By conjugating leptin to liposomes, L-LP-Fi/CeNPs specifically target DPCs that express LEPR, resulting in a 167.9% increase in drug retention in the skin and a 13.4% increase in cellular uptake within 6 h (Fig. 1A). Upon reaching target cells, finasteride and CeNPs coordinately modulate ROS and DHT levels, subsequently down-regulating the p16/pRb pathway expression to inhibit DPC senescence while preserving their secretory function (Fig. 1B).

In the AGA mouse model, L-LP-Fi/CeNPs demonstrated therapeutic efficacy comparable to, or modestly improved over, that of minoxidil in terms of follicular microenvironment remodeling, anagen phase transition, and hair regeneration. Overall, this study presents an LEPR-targeted nanomodulation strategy that inhibits premature DPC senescence through dual regulation of androgen signaling and oxidative stress, offering an effective and mechanistically targeted hair regeneration approach for AGA in preclinical models. (For a full list of abbreviations, see Table S1.)

## Materials and Methods

### Materials

Lecithin (Chemical Abstracts Service [CAS] No. 8002-43-5) and cholesterol (CAS No. 57-88-5) were sourced from Sigma-Aldrich (USA). Cerium acetate (CAS No. 206996-60-3), oleylamine (CAS No. 112-90-3), *N*-distearoylphosphatidylacetamide polyethylene glycol-activated ester (DSPE–PEG–NHS; BIO-000001), and androst-4-en-3-one (T, CAS No. 58-22-0) were obtained from Aladdin Industry, Inc. (Shanghai, China). Xylene (CAS No. 1330-20-7) was acquired from Sinopharm Chemical Reagent Co., Ltd. (Shanghai, China). Finasteride (CAS No. 98319-26-7) was purchased from Shanghai Macklin Biochemical Technology Co., Ltd. (Shanghai, China). Leptin protein (HY-P70704A) was obtained from MedChemExpress (USA). The anti-LEPR antibody (ET1704-44) was acquired from HUABIO (Hangzhou, China). Cell Counting Kit-8 (CCK-8) was sourced from Meilunbio Biotechnology Co., Ltd. (Dalian, China). The human transforming growth factor-β1 (TGF-β1) enzyme-linked immunosorbent assay (ELISA) kit (MM-0090H2) and human β-catenin ELISA kit (MM-2451H2) were purchased from Jiangsu Meimian Industrial Co., Ltd. (Jiangsu, China). The total SOD assay kit (S0101S), senescence β-galactosidase staining kit (C0602), ROS assay kit (S0033S), cell plasma membrane staining kit with 1,1′-dioctadecyl-3,3,3′,3′-tetramethylindocarbocyanine perchlorate (DiI) (C1991S), and 4′,6-diamidino-2-phenylindole (DAPI; C1002) were obtained from Shanghai Beyotime Biotechnology Co., Ltd. (Shanghai, China). p53 (A0263), horseradish peroxidase (HRP) goat anti-rabbit IgG(H+L) (AS014), and HRP goat anti-mouse IgG(H+L) (AS003) were acquired from ABclonal Technology Co., Ltd. (Wuhan, China). The p16 antibody (10883-1-AP), pRb antibody (30376-1-AP), and β-actin (66009-1-Ig) were sourced from Proteintech Group, Inc. (China). CD31 (ab281583) and SRY-box transcription factor 9 (SOX9; ab185966) were purchased from Abcam (UK). Ki67 (CST9129) was obtained from Cell Signaling Technology, Inc. (USA).

### Cell culture

Human dermal papilla cells (HDPCs) were isolated by Zhejiang Meisen CTCC Cellular Technology Co., Ltd on 2022 March 12. Cell identity was confirmed by positive immunofluorescence staining for alpha-smooth muscle actin, a characteristic marker of DPCs. The human immortalized keratinocyte cell line HaCaT (RRID: CVCL_0038) was obtained from the same provider on 2021 May 22, with authentication performed by short tandem repeat profiling matching reference standards. The mouse dermal papilla cells (MDPCs) were isolated by Jiangsu Meimian Industrial Co., Ltd. on 2023 April 26 and authenticated as a DPC cell line through alpha-smooth muscle actin immunofluorescence staining. Mouse epidermal keratinocytes (mEKs) (RRID: CVCL_Z980) were obtained from the same vendor on 2024 April 10, with >90% purity confirmed by pan-cytokeratin immunofluorescence. Each cell line tested negative for mycoplasma contamination using the MycoAlert detection kit (Lonza) and showed no microbial contamination during 14-d sterile culture monitoring. HDPCs and HaCaT cells were cultured in Dulbecco’s modified Eagle medium (Gibco, USA) supplemented with 10% fetal bovine serum, 100 μg/ml streptomycin (Gibco, USA), and 100 U/ml penicillin (Gibco, USA). MDPCs and mEKs were cultured in a complete medium supplied by the aforementioned companies.

### Validation of LEPR expression in DPCs

Cell immunofluorescence and Western blot analyses were employed to assess LEPR expression. Initially, HDPCs, HaCaT cells, MDPCs, and mEK cells were seeded at a density of 5 × 10^5^ cells into 12-well plates containing crawler sheets and allowed to adhere overnight. Following this, the cells were fixed with 4% paraformaldehyde for 10 min and subsequently incubated with primary and secondary antibodies, as well as DAPI. The samples were then photographed using a confocal laser scanning microscope (CLSM; IX83-FV3000-OS, Olympus, Japan) for semiquantification of LEPR protein expression. Proteins were quantified using the bicinchoninic acid (BCA) assay after extracting cellular proteins from HDPC and HaCaT cells. Equal amounts of protein samples were subjected to sodium dodecyl sulfate–polyacrylamide gel electrophoresis (SDS-PAGE) for separation. Protein samples were then transferred onto polyvinylidene fluoride membranes under a constant current of 300 mA. The membranes were blocked with 5% nonfat skim milk in 1× TBST (10 mM Tris–HCl, 150 mM NaCl, and 0.05% Tween 20) at room temperature for 2 h, followed by incubation with primary antibodies at 4 °C overnight. Afterward, the membranes were incubated with HRP-conjugated anti-immunoglobulin G (anti-IgG) secondary antibody for 2 h and visualized using an enhanced chemiluminescence (ECL) detection kit. LEPR expression was quantified using the ImageJ software after exposure to Bio-Rad chemiluminescence.

### In vitro cell colocalization of leptin

To clarify the colocalization of LEPR with leptin, imaging was conducted using a CLSM (IX83-FV3000-OS; Olympus, Japan). HDPCs were cultured in 12-well plates containing coverslips. Following cell adhesion, 10 μg of fluorescein isothiocyanate (FITC)-labeled leptin (10:1, w/w) was introduced into the medium. The cells were subsequently fixed in a sterile medium. Fixation was achieved using 4% paraformaldehyde, and cells were permeabilized with 0.1% Triton X-100. Intracellular LEPR was labeled with a rabbit anti-LEPR protein monoclonal antibody (1:100, HUABIO), followed by an Alexa Fluor 561 goat anti-rabbit secondary antibody incubation. Cellular imaging was then recorded using a CLSM with a red/green fluorescence exciter.

### Synthesis and characterization of CeNPs

CeNPs were prepared using the inverse micelle method. First, 0.43 g of cerium acetate was stirred in 3.14 g of oleylamine and 15 ml of xylene at room temperature for 24 h. Subsequently, the mixture was heated to 90 °C in a water bath under an argon atmosphere. Upon clarification of the solution, 1 ml of deionized water was added promptly, and the mixture was stirred for another 3 h until the liquid was clarified again. After cooling, CeNPs were added to 50 ml of ethanol and washed 3 times with ethanol. The resulting CeNPs were dispersed in trichloromethane and stored at −20 °C, shielded from light.

The morphology of the CeNPs was characterized using transmission electron microscopy (TEM). X-ray photoelectron spectroscopy (XPS) was performed using a Thermo Scientific ESCALAB 250 Xi XPS instrument to acquire the spectra. The SOD mimetic activity (SOD−) of CeNPs at concentrations of 10, 20, 30, and 40 μg/ml was assessed using an SOD assay kit, following the manufacturer’s protocol. Additionally, CAT activity was evaluated with a dissolved oxygen meter by mixing 0.1 M H_2_O_2_ with varying concentrations of CeNPs (10, 20, 30, and 40 μg/ml) in 15 ml of deionized water, and the resulting oxygen production was recorded using the oxygen electrode of an AR-8406 dissolved oxygen meter. The long-term redox stability of CeNPs was evaluated by comparing their SOD and CAT mimetic activities at a concentration of 20 μg/ml on day 1 and after 21 d of storage. Additionally, the valence cycling capacity was assessed by adding 10 μl of H_2_O_2_ to a 1 mg/ml CeNP solution, and the repeated valence transition cycle was recorded over a 14-d period.

### Fabrication and characterization of leptin-modified co-loaded liposomes

Leptin-functionalized, co-loaded liposomes (L-LPs) were prepared using the thin-film hydration technique. Briefly, cholesterol, lecithin, and DSPE–PEG–NHS were combined at a mass ratio of 9:3:0.1 in chloroform. Finasteride and CeNPs were co-dissolved in the organic phase at a mass ratio of 0.75:0.5. Chloroform was evaporated under reduced pressure using a rotary evaporator to form a thin lipid film on the wall of a round-bottom flask. The film was then hydrated with phosphate-buffered saline (PBS) under gentle agitation. The resulting multilamellar vesicle suspension was subjected to probe sonication on an ice bath (15 min, 480 W, pulsed mode: 3 s on, 2 s off) to obtain small unilamellar liposomes. The final liposomal suspension had a total lipid concentration of 1.0 mg/ml. For surface functionalization, the liposomes were incubated with leptin (final concentration: 100 μg/ml) at 4 °C for 12 h under constant stirring to form L-LPs.

The liposomes underwent comprehensive characterization, including assessments of morphology, size distribution, zeta potential, drug-loading capacity, and release profile. The size distribution and zeta potential of the liposomes (L-LPs) were evaluated using dynamic light scattering with a Litesizer 500 instrument (Anton-Paar, Austria). Morphological analysis was conducted via TEM using a JEM-1400 microscope (JEOL, Japan) following negative staining with a 1% (w/v) phosphotungstic acid solution. The encapsulation efficiency of finasteride and CeNPs within the L-LPs was quantified using high-performance liquid chromatography (HPLC) and inductively coupled plasma–mass spectrometry, respectively. Initially, unencapsulated finasteride was separated from the L-LPs by centrifugation at 4,500 g for 10 min in an ultrafiltration tube (10,000 Da; Millipore). The amount of drug in the filtrate was then quantified using an HPLC system (Agilent 1200, USA) under the following parameters: column, ZORBAX SB-C18 column; mobile phase, acetonitrile–water (50:50, v/v); flow rate, 1.0 ml/min; and detection wavelength, 210 nm. Additionally, dialysis was used to assess the in vitro release profile of finasteride. Briefly, 0.5 ml of newly synthesized L-LPs with a molecular weight cutoff of 5,000 Da was placed in a dialysis bag, submerged in 6.0 ml of various release media containing 0.1% Tween 20, and shaken at 150 rpm at 37 °C. The release medium was periodically replaced with a fresh heated solution. HPLC analysis was then used to ascertain the drug content of the release medium.

### In vitro assessment of skin penetration and skin retention

In vitro transdermal experiments were conducted utilizing a Franz diffusion cell to simulate skin permeation. The receiving chamber was filled with PBS solution, which was continuously stirred at a constant rate of 200 rpm. The temperature of the diffusion cell was meticulously maintained at 37 °C. A suitably sized section of porcine skin was securely positioned between the donor and receiver chambers. Subsequently, the donor chamber was filled with 1 ml of an equivalent concentration of LPs and L-LPs, which were stained with 1 μl of DiI, and then sealed with a sealing membrane. The reaction was conducted for 12 and 24 h, respectively, while being shielded from light. Subsequently, the porcine skin samples were cryogenically preserved using liquid nitrogen, and the resulting sections were imaged utilizing a CLSM (IX83-FV3000-OS, Olympus, Japan). The in vitro skin retention assay employed a Franz diffusion cell to replicate skin retention conditions. Under the aforementioned parameters, equivalent quantities of free finasteride, LP-Fi, and L-LP-Fi (each containing 50 μg of finasteride) were administered into the donor compartment of the Franz diffusion cell, which was then sealed with a membrane. At predetermined intervals, equal volumes of the transdermal permeate solution were collected and a fresh buffer solution was replenished in the donor compartment. After 24 h, the pig skin was excised, and 3 ml of PBS was added to facilitate the grinding and extraction of the retained drug. The concentration of the drug concentration was subsequently quantified using the HPLC method previously described.

### In vitro colocalization of L-LPs in skin

The colocalization of LEPR and L-LPs was assessed through a skin penetration simulation using a Franz diffusion cell. In this experimental design, 1 ml of DiI-stained L-LPs (incorporating 1 μl DiI) was administered to the donor chamber, with mouse skin positioned between the donor and recipient chambers. Following a 24-h incubation, the mouse skin was subjected to cryosectioning and subsequently labeled with a rabbit monoclonal antibody specific to LEPR proteins (dilution 1:100; HUABIO), followed by incubation with an Alexa Fluor 488-conjugated goat anti-rabbit secondary antibody. The resulting tissue samples were then analyzed using a CLSM (IX83-FV3000-OS, Olympus, Japan) with red/green fluorescence excitation.

### Evaluation of cell safety and concentration screening

To ascertain the safe concentration of the drug, HDPCs in the logarithmic growth phase were seeded into 96-well plates at a density of 5 × 10^3^ cells per well in 100 μl of culture medium. Following a 24-h period for cell attachment, 5 replicate wells were designated for each treatment group and incubated overnight at 37 °C in a 5% CO_2_ environment. To evaluate the combined effects of Fi and CeNPs, a matrix concentration design was employed. Fi (0, 2, 4, 6, and 8 μg/ml) and CeNPs (0, 2, 4, 6, and 8 μg/ml) were systematically crossed in all possible combinations, and the resulting effects on cell viability were measured for each group. Control wells containing no drug treatment were also included and incubated for 12 h. Subsequently, 10 μl of the CCK-8 reagent was introduced to each well for 1 h. The luminescence of the samples was quantified using a microplate reader set to an excitation wavelength of 450 nm. Cell viability was calculated using the formula

$$\text{Cell viability}\;\left(\%\right)=\frac{A_{\text{sample}}-A_{\text{blank}}}{A_{\text{control}}-A_{\text{blank}}}\times 100\%$$ (1)

To identify the optimal therapeutic concentration of the drugs and to assess the synergistic effects of the 2 drugs under T-induced modeling conditions, cells were plated as previously described. Various concentrations of the drugs (Fi: 0, 2, 4, 6, and 8 μg/ml; CeNPs: 0, 2, 4, 6, and 8 μg/ml) were added to each well and incubated for 12 h, followed by an additional 12-h incubation with T. The model group that did not receive drug treatment served as the control for evaluating the optimal growth concentration of the cells.

$$\text{Cell viability}\;\left(\%\right)=\frac{A_{\text{sample}}-A_{\text{blank}}}{A_{\text{model}}-A_{\text{blank}}}\times 100\%$$ (2)

Following drug combination treatment, the interaction effects were quantitatively analyzed using multiple models. Specifically, the Bliss independence model, the highest single agent model, and the Loewe additivity model were applied to assess the combined effects across the different concentration pairs. All analyses were performed in the R environment using relevant packages for model fitting and synergy score calculation. Based on the criteria of each model, an observed effect greater than the predicted value was defined as synergy, an observed effect equal to the predicted value indicated an additive effect, and an observed effect less than the predicted value suggested antagonism.

### Cellular uptake

The investigation of the ability of HDPCs to internalize L-LPs was conducted using a CLSM (IX83-FV3000-OS, Olympus, Japan) and flow cytometry (FCM; ACEA NovoCyte; ACEA Biosciences, USA). HDPCs were seeded at a density of 1 × 10^5^ cells into 12-well plates containing coverslips and were allowed to adhere overnight. Subsequently, DiI-labeled L-LPs were introduced at a dilution of 1:1,000 (v/v), corresponding to a concentration of 25 μg/ml, with the same concentration of LPs utilized as a control. The plates were incubated at 37 °C for 2 and 6 h. Fluorescence intensity was quantified using a CLSM at both time points posttreatment. Additionally, HDPCs were seeded at the same density into 12-well plates, allowed to adhere overnight, and then treated with the same concentration of DiI-labeled L-LPs (1:1,000, v/v) across 3 replicate wells. The cells were cultured at 37 °C for 2 and 6 h with LPs serving as a control. FCM was subsequently employed to evaluate the fluorescence intensity of cellular uptake of L-LPs after the 2- and 6-h treatment periods, thereby elucidating the uptake capacity of HDPCs for L-LPs.

### Assay for senescence-associated β-galactosidase activity

HDPCs were seeded into 6-well plates at a density of 5 × 10^5^ cells per well and cultured overnight. Each well was treated with drugs (Fi/CeNPs, L-LP-Fi, L-LP-CeNPs, LP-Fi/CeNPs, and L-LP-Fi/CeNPs; with a concentration Fi at 2.0 μg/ml and that of CeNPs at 2.0 μg/ml) for 12 h, followed by treatment with T (concentration: 30 μM) for another 12 h. The cells were then fixed using 4% paraformaldehyde for 10 min at room temperature. The subsequent experiments were conducted in accordance with the protocol provided with the senescence-associated β-galactosidase (SA-β-gal) staining kit (Biotechnology, Shanghai, China). The presence of SA-β-gal-positive cells, which appeared blue, was assessed using an inverted microscope (Nikon Eclipse TS100, Nikon, Japan), and the mean percentage of SA-β-gal-positive cells was calculated.

### Measurement of intracellular ROS

The levels of intracellular ROS were quantified using FCM (ACEA NovoCyte; ACEA Biosciences, USA) according to the following protocol: HDPCs were inoculated into 12-well plates at a density of 1 × 10^5^ cells per well and cultured overnight. Subsequently, each well was treated with various drug formulations (Fi/CeNPs, L-LP-Fi, L-LP-CeNPs, LP-Fi/CeNPs, and L-LP-Fi/CeNPs; with a concentration of Fi at 2.0 μg/ml and that of CeNPs at 2.0 μg/ml) for 6 h, followed by exposure to T (concentration: 30 μM) for 2 h. Upon completion of the pretreatment, the HDPCs were incubated with 2′,7′-dichlorodihydrofluorescein diacetate (1 μM) for 10 min at 37 °C in light-protected conditions. After centrifugation, the cells were washed twice with PBS and then analyzed by FCM. The results were obtained by assessing the percentage of fluorescent cells.

### Expression of senescence-related proteins

Western blot analysis was used to detect the expression levels of p16/pRb and p53. HDPCs were seeded into 6-well plates at a density of 1 × 10^5^ cells per well and cultured overnight. Each well was then treated with various drugs (Fi/CeNPs, LP-Fi/CeNPs, and L-LP-Fi/CeNPs) at a concentration of 2.0 μg/ml for both Fi and CeNPs for 12 h, followed by treatment with T at a concentration of 30 μM for an additional 12 h. The cells were then lysed using radioimmunoprecipitation assay buffer supplemented with a protease inhibitor and incubated on ice for 15 min. The resulting cell lysates were centrifuged at 12,000 rpm for 20 min at 4 °C. Following centrifugation, the protein concentration was quantified using the Pierce BCA protein assay kit (Biotechnology, Shanghai, China) at a wavelength of 562 nm. Equal amounts of protein (10 μg) were subjected to SDS-PAGE using a 4% to 20% polyacrylamide gel. The proteins were subsequently transferred to a polyvinylidene fluoride membrane at a constant voltage, with p16 being transferred for 30 min, and pRb and p53 were transferred for 45 min. The membranes were then blocked with 5% nonfat skim milk in 1× TBST (10 mM Tris–HCl, 150 mM NaCl, and 0.05% Tween 20) at room temperature for 2 h, followed by incubation with primary antibodies at 4 °C overnight. Afterward, the membranes were incubated with HRP-conjugated anti-IgG secondary antibodies for 1 h, and protein bands were visualized using an ECL detection kit (Bio-Rad).

### ELISA for TGF-β1 and β-catenin

To quantify TGF-β1 and β-catenin in the conditioned medium derived from DPCs, the human TGF-β1 ELISA kit and the human β-catenin ELISA kit, both sourced from Meimian (China), were employed. HDPCs were seeded into 12-well plates at a density of 1 × 10^5^ cells per well and cultured overnight. Each well was then incubated with various drugs (Fi/CeNPs, L-LP-Fi, L-LP-CeNPs, LP-Fi/CeNPs, and L-LP-Fi/CeNPs) at a concentration of 2.0 μg/ml for both Fi and CeNPs for 12 h. Following this, the cells were exposed to T (concentration: 30 μM) for another 12 h. The supernatants and cell lysates were then collected separately, and the levels of TGF-β1 and β-catenin were measured using the ELISA kits following the manufacturer’s instructions.

### Development of the in vivo animal study

The hair regeneration potential of L-LP-Fi/CeNPs was assessed in male C57BL/6 mice, aged 6 weeks, sourced from Shanghai SLAC Laboratory Animal Co. Ltd. (Shanghai, China). In adherence to ethical standards for animal welfare, all experimental procedures received approval from the animal ethics committee of Zhejiang University and complied with the institution’s guidelines. To develop a mouse model of AGA, a 1 cm × 1.5 cm section of dorsal skin from the resting C57BL/6 mice was depilated. The mice were then randomly divided into 8 groups (*n* = 6 per group): control group, model group, minoxidil group, Fi/CeNP group, L-LP-Fi group, L-LP-CeNP group, LP-Fi/CeNP group, and L-LP-Fi/CeNP group. The AGA model was induced through the topical administration of a T solution (0.5% w/v) in ethanol (1:1, v/v) at a dosage of 0.1 ml cm^−2^ for 21 d. Treatment commenced on the first day post-epilation, with the application of the drug at a volume of 0.15 ml cm^−2^. The positive control group received a daily topical application of a 5% minoxidil solution (0.15 ml cm^−2^). Digital images of the depilated area were captured on days 1, 7, 14, 18, and 21. On day 21 following epilation, the diameter of the regrown hairs was evaluated using scanning electron microscopy (SEM; SU-8010, Japan), and the coverage of the regrowing hairs was quantified utilizing the ImageJ software.

### Histological staining of skin samples

On day 14, the mice were euthanized to facilitate the staining of the depilated skin areas using hematoxylin–eosin (H&E) and to assess the expression levels of dihydroethidium (DHE), Ki67, CD31, SOX9, and p16. Skin samples were fixed in 4% w/v formaldehyde for subsequent paraffin embedding. Following dehydration, the tissues were embedded in paraffin and sectioned into slices of 3- to 4-μm thickness. The sections underwent dewaxing and rehydration prior to staining with hematoxylin for 5 min and eosin for 1 min. A subset of H&E-stained sections was also scanned. Additionally, tissues were frozen, embedded in an optimal cutting temperature compound, and sectioned into 10-μm slices utilizing a cryostat technique. These sections were imaged after treatment with DAPI (Roche, USA) and 2.5 mM DHE (Sigma, USA). The deparaffinized and rehydrated tissue slices were then incubated with primary antibodies against Ki67 (CST, USA), CD31 (Abcam, USA), SOX9 (Abcam, USA), and p16 (Abcam, USA) antibodies for immunofluorescence analysis. Following this, the sections were treated with the corresponding fluorescently labeled secondary antibodies, and the cell nuclei were stained with DAPI.

### ELISA analysis for TGF-β1 and β-catenin levels

Upon euthanization, skin samples were collected and obtained from designated hair removal areas at specified time intervals corresponding to the various experimental groups. The collected skin samples were homogenized through mechanical grinding with a steel ball in PBS solution, followed by centrifugation at 15,000 rpm for 20 min. The resulting supernatant was subsequently analyzed for concentration using a specific assay kit.

### Statistical analysis

All data are presented as mean with standard deviation. Statistical analyses, including the unpaired *t* test, one-way analysis of variance, and correlation analyses, were performed using GraphPad Prism version 10.0. A *P* value of less than 0.05 was deemed statistically significant.

## Results and Discussion

### Identification of LEPR expression in DPCs and validation of leptin binding

**Fig. 2.**
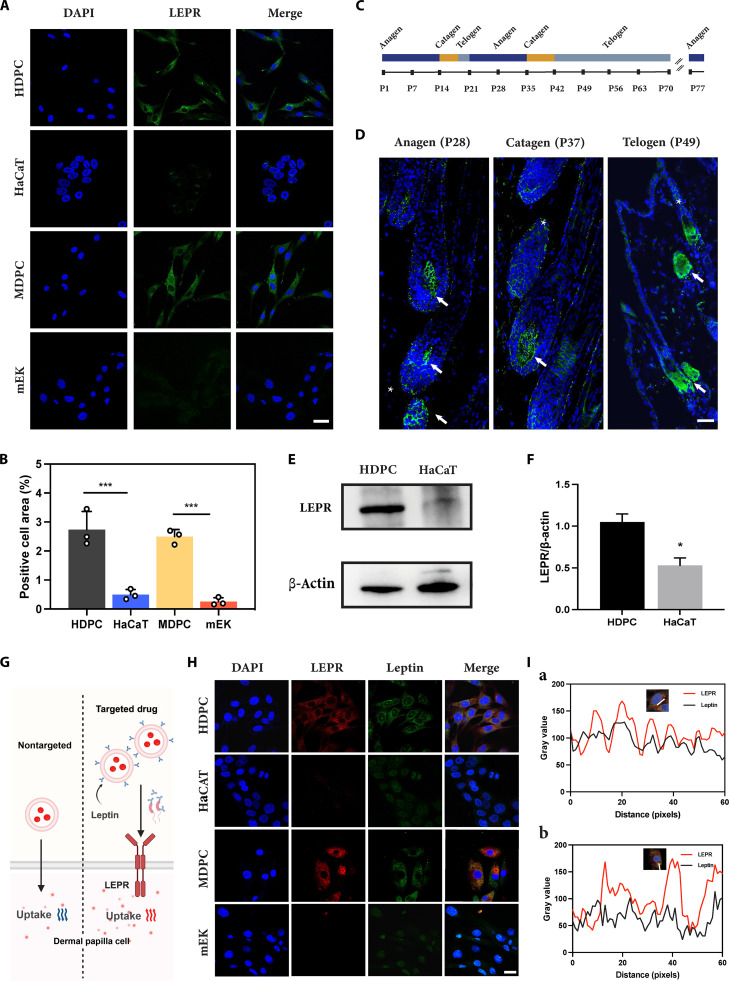
LEPR expression in DPCs and validation of binding to leptin. (A) LEPR expression in HDPCs, HaCaT cells, MDPCs, and mEK cells. Scale bar = 50 μm. (B) Quantitative analysis of LEPR expression in cells (*n* = 3). (C) Time scale analysis of the HF cycle in C57BL/6 mice at 11 weeks after birth. (D) LEPR expression in different HF phases of mice. Scale bar = 25 μm. (E and F) Western blotting and grayscale analysis of LEPR expression (HDPC and HaCaT) (*n* = 3). (G) The mechanism by which leptin targets LEPR-expressing DPCs. (H) CLSM imaging following co-incubation with FITC-labeled leptin (green) and Alexa Fluor 647-labeled LEPR (red). Scale bar = 20 μm. (I) Grayscale colocalization analysis of the images shown in (H), (a) PCC_(HDPC)_ = 0.5298 and (b) PCC_(MDPC)_ = 0.5263. All results are presented as means ± SDs (*P < 0.05 and ***P < 0.001). DAPI, 4′,6-diamidino-2-phenylindole; HF, hair follicle; HDPC, human dermal papilla cell; MDPC, mouse dermal papilla cell; mEK, mouse epidermal keratinocyte; FITC, fluorescein isothiocyanate; PCC, Pearson correlation coefficient.

Recent advancements in single-cell transcriptomics have identified LEPR as a hallmark gene in DPCs [30–32]. To further explore and investigate LEPR as a potential therapeutic target, we performed immunofluorescence staining, utilizing keratinocyte cell lines (HaCaT and mEK) as controls, alongside HDPCs and MDPCs. The findings revealed a pronounced expression of LEPR in both HDPCs and MDPCs, while keratinocytes exhibited comparatively lower levels of expression (Fig. 2A and B). Based on the postnatal HF cycle in mice (Fig. 2C) [33], we conducted a screening for HFs during the anagen (P28), catagen (P37), and telogen (P49) phases for immunofluorescence analysis. LEPR expression was robustly detected in DPCs across all HF cycle phases (Fig. 2D, arrows), whereas only isolated positive cells were noted outside of the HFs (Fig. 2D, asterisks). This expression pattern, which is an independent expression pattern of the HF cycle, highlights the stability of LEPR as a target. Additionally, Western blot analysis provided qualitative confirmation of the protein expression results, aligning with the immunostaining data, as LEPR was identified in HDPCs (Fig. 2E and F). These observations substantiate the potential of LEPR as a target for therapeutic strategies directed at DPCs.

Leptin, a 16-kDa cytokine primarily secreted by adipose tissue, exhibits a strong affinity for LEPR and is capable of crossing the blood–brain barrier to bind to LEPR [34]. In light of this, we investigated the feasibility of utilizing leptin for effective targeting of DPCs (Fig. 2G). To assess the targeting capability of leptin, it was conjugated with FITC, while LEPR was labeled with Alexa Fluor 647. The intracellular fluorescence of both conjugates was subsequently evaluated using a CLSM. The results demonstrated dual fluorescence colocalization of leptin and LEPR in both HDPCs and MDPCs (Fig. 2G). Furthermore, Pearson correlation coefficient analysis revealed a strong linear correlation between the 2, indicating a specific binding interaction between leptin and LEPR (Fig. 2H).

### Synthesis and characterization of CeNPs

**Fig. 3.**
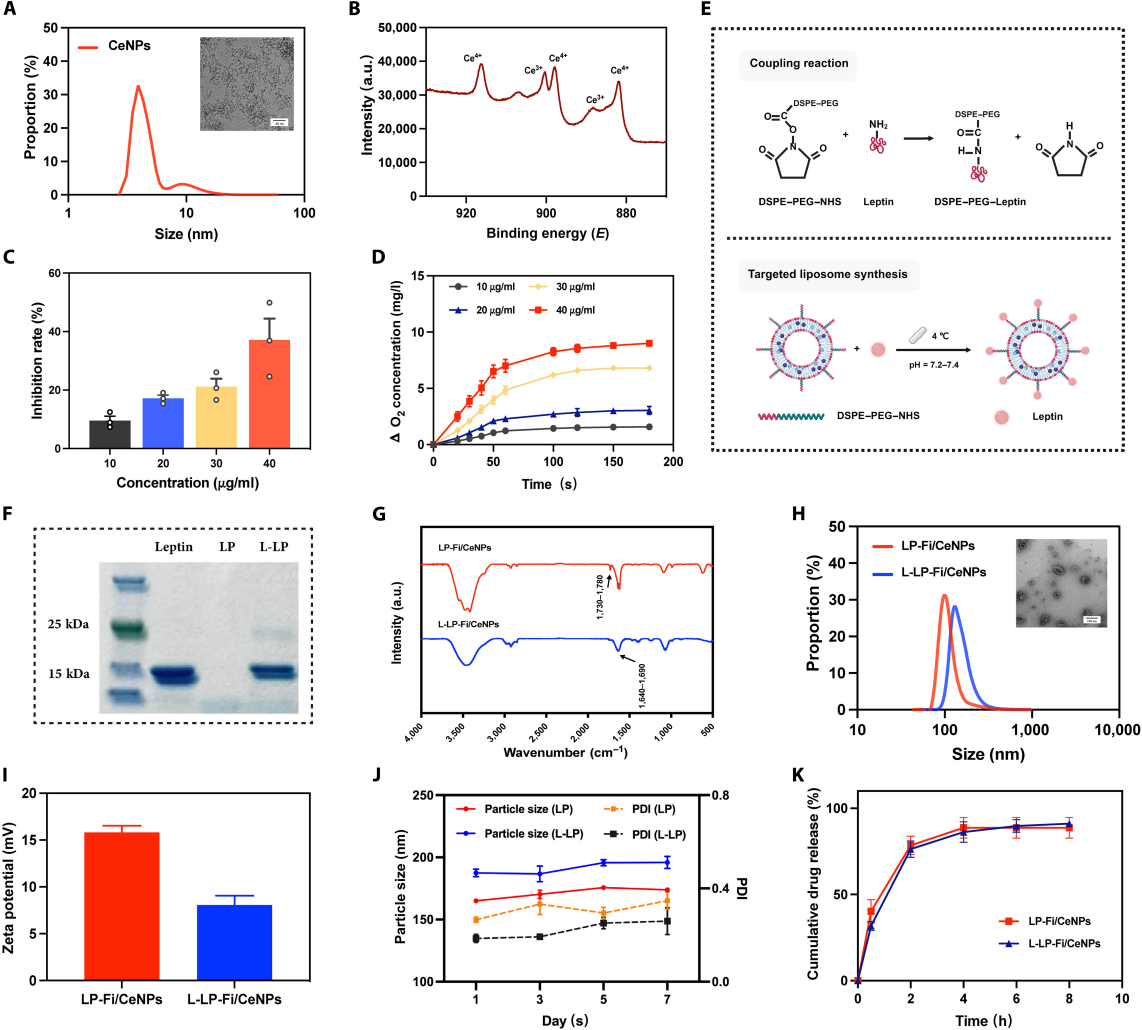
Preparation and characterization of CeNPs and L-LP-Fi/CeNPs. (A) Particle size distribution and TEM images of CeNPs in chloroform. Scale bar = 20 nm. (B) XPS of CeNPs. (C) Superoxide (SOD)-like activity of CeNPs (*n* = 3). (D) Catalase (CAT)-like activity of CeNPs (*n* = 3). (E) Schematic illustration of the fabrication process of L-LP-Fi/CeNPs. (F) SDS-PAGE of leptin, LP, and L-LP. (G) Infrared spectra of LP and L-LP. (H) Particle size distribution and TEM image of LP-Fi/CeNPs and L-LP-Fi/CeNPs. Scale bar = 100 nm. (I) Zeta potentials of LP-Fi/CeNPs and L-LP-Fi/CeNPs. (J) LP-Fi/CeNP and L-LP-Fi/CeNP particle size and PDI in PBS over time. (K) Release profile of LP-Fi/CeNPs and L-LP-Fi/CeNPs. The data are expressed as means ± SDs (*n* = 3). TEM, transmission electron microscopy; XPS, x-ray photoelectron spectroscopy; SDS-PAGE, sodium dodecyl sulfate–polyacrylamide gel electrophoresis; DSP, *N*-distearoylphosphatidylacetamide; NHS, *N*-hydroxysuccinimide; PDI, polydispersity index.

The synthesis of CeNPs was conducted utilizing the previously established reverse micelle approach [21]. TEM analysis indicated that the CeNPs exhibited an average diameter of 7.7 ± 1.4 nm and maintained a uniform morphology (Fig. 3A). Furthermore, XPS analysis (Fig. 3B) identified distinct peaks corresponding to Ce (approximately 900 eV), indicating that the CeNPs consist of Ce ions in 2 oxidation states: Ce^3+^ and Ce^4+^. The coexistence of these 2 oxidation states is fundamental to the redox functionality of CeNPs, establishing the structural basis for enzyme-mimetic catalytic activities [35]. Subsequently, the enzyme-mimetic activities of CeNPs, specifically their SOD and CAT mimetic activities, were evaluated. The findings demonstrated that CeNPs effectively scavenged superoxide anions in a concentration-dependent manner (Fig. 3C) and facilitated the conversion of H_2_O_2_ to oxygen (Fig. 3D), thereby confirming their capacity to mitigate ROS.

To evaluate the long-term stability of the redox capacity of CeNPs, we measured their CAT and SOD activities on day 21, along with their valence transition behavior. The results showed that detectable CAT and SOD activities were still maintained in CeNPs at this time point (Fig. S1A and B), indicating that their redox functionality was preserved throughout the experimental period. Furthermore, the CeNPs exhibited reversible color changes upon reaction with H_2_O_2_ (Fig. S1C): The solution turned from pale yellow to orange, corresponding to the oxidation of Ce^3+^ to Ce^4+^, and then gradually reverted to pale yellow, reflecting the reduction of Ce^4+^ back to Ce^3+^. These findings collectively demonstrate that the CeNPs retained a reversible valence transition capability and sustained redox activity over the 14-d experimental cycle.

### Preparation and characterization of L-LP-Fi/CeNPs

In order to facilitate the attachment of leptin to the surface of liposomes, we utilized the *N*-hydroxysuccinimide (NHS) moiety present in 1,2-distearoyl-*sn*-glycero-3-phosphoethanolamine (DSPE–PEG–NHS). This approach enables the formation of a stable and irreversible amide bond through the amidation of the primary amine group in leptin. The esterification reaction promotes the rapid conjugation of leptin to the liposomal structure (Fig. 3E). Utilizing the thin-film dispersion technique, we successfully synthesized liposomes with DSPE–PEG–NHS affixed to the surface, encapsulating both finasteride and CeNPs within. Subsequently, we generated leptin-modified targeting liposomes via the aforementioned reaction. To confirm the effective conjugation of leptin, we employed SDS-PAGE and infrared spectroscopy to assess the protein expression on the liposome surface. The SDS-PAGE analysis indicated a shift in the mobility and positional delay of the leptin–liposome (L-LP) conjugate relative to native leptin, indicating successful conjugation (Fig. 3F). Furthermore, infrared spectroscopy demonstrated a reduction in the peak near 1,780 cm^−1^, which is characteristic of the NHS ester, diminished due to the transformation of functional groups pre- and post-reaction. Concurrently, a new amide I band peak emerged, transitioning from 2 distinct peaks to a singular peak, further indicating the effective conjugation of leptin to the liposome (Fig. 3G).

Subsequently, TEM analysis revealed that the synthesized liposomes exhibited a typical saucerlike morphology and were uniformly distributed (Fig. 3H). Both LP-Fi/CeNPs (162.6 ± 1.4 nm) and L-LP-Fi/CeNPs (188.4 ± 2.8 nm), as measured by dynamic light scattering, fell within the 200-nm size range (Fig. 3H). These measurements confirm the physicochemical characteristics of the prepared liposomes used in subsequent experiments [36]. In terms of zeta potential, the L-LP-Fi/CeNPs (8.5 ± 0.5 mV) presented a reduced positive charge compared with the LP-Fi/CeNPs (15.9 ± 0.7 mV) (Fig. 3I), which was likely attributable to the negative charge imparted by leptin. Despite this reduction, L-LP-Fi/CeNPs retained a net positive charge, allowing for effective interaction with negatively charged cell membranes through electrostatic attraction. Furthermore, the liposome particle size and polydispersity index (PDI) of the liposomes were monitored over a period of 7 d, revealing no significant changes (Fig. 3J). Extended stability profiling over 21 d confirmed the robustness of the L-LP-Fi/CeNP formulation. No significant variations in particle size or PDI were observed at storage temperatures of 4 and 25 °C (Table S2), and TEM imaging corroborated these findings by verifying the preserved structural integrity of the liposomes (Fig. S2). These findings indicate that the synthesized L-LP-Fi/CeNPs exhibit stability, thereby supporting their subsequent application in both in vitro and in vivo studies.

The encapsulation and loading efficiencies for finasteride were determined to be 65.26% and 2.50%, respectively, while the CeNPs demonstrated encapsulation and loading efficiencies of 77.5% and 3.1%, respectively. The release profile of finasteride in the presence of 1% Tween, pH = 7.4 in PBS, indicated a release 90.0% within 8 h (Fig. 3K). These results are consistent with previous studies and may be attributed to the diminished stability of cationic liposomes in an alkaline condition, which accelerates the release of the drug from the liposomal carriers [37,38].

### Transdermal retention and cellular uptake of L-LP

**Fig. 4.**
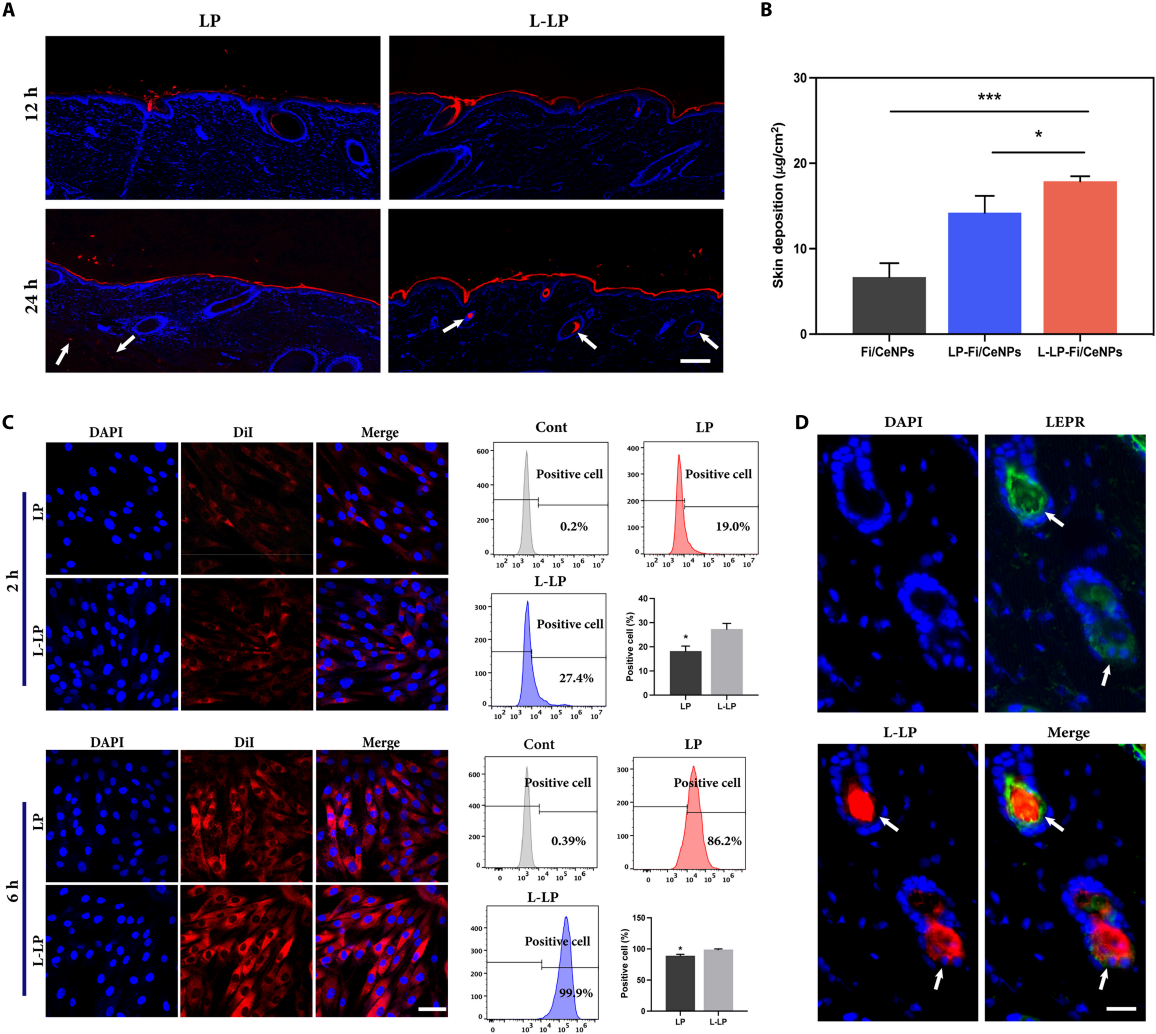
Transdermal retention and cellular uptake of L-LPs. (A) CLSM images of pig skin after the application of 1,1′-dioctadecyl-3,3,3′,3′-tetramethylindocarbocyanine perchlorate (Dil)-labeled LPs or L-LPs for 12 and 24 h. Scale bar = 200 μm. (B) In vitro skin retention of liposomes and free drug (*n* = 3). (C) FCM analysis and CLSM images of DPCs exposed to LPs or L-LPs for 2 or 6 h. Scale bar = 50 μm (*n* = 3). (D) Colocalization images of LEPR and L-LPs. Scale bar = 100 μm (*P < 0.05 and ***P < 0.001). CLSM, confocal laser scanning microscope; FCM, flow cytometry.

The penetration dynamics of LP and L-LP in isolated skin over 12 and 24 h demonstrated that L-LPs elicited a more pronounced fluorescence within HFs (Fig. 4A). The HF structures were marked with blue fluorescence, while the liposomes, labeled with DiI, exhibited red fluorescence. After 12 h, liposomes from both the LP and L-LP groups were primarily localized within the epidermal stratum corneum and superficial dermal follicles, reaching a penetration depth of approximately 200 to 250 μm. By the 24-h mark, red fluorescence was observed extending into the dermis and, in the LP group, into the subcutaneous tissue, with penetration depths of approximately 250 to 700 μm. This distribution indicates a time-dependent transdermal delivery process, with liposomes retained across multiple skin layers.

At 24 h, the red fluorescence in the LP group was dispersed across all tissue layers, exhibiting weak fluorescence in the dermis and HFs, alongside detectable fluorescence in the subcutaneous tissue (Fig. 4A, arrows). Conversely, the L-LP group displayed concentrated red fluorescence within the HFs in the dermis, with no fluorescence detected in the subcutaneous tissue. This distribution pattern suggests preferential localization within the targeted treatment area via the follicular pathway, with reduced diffusion into subcutaneous tissues.

In vitro retention assays demonstrated that liposomes substantially enhanced the transdermal drug delivery rate compared to the free drug solution. The skin retention assessment (Fig. 4B) showed that drug retention in the skin increased by 129.2% and 167.9% for LP (Fi) and L-LP (Fi), respectively, compared with that of the free Fi solution (6.68 vs. 15.31 vs. 17.89 μg/cm^2^). The efficacy of drug uptake by DPCs was further substantiated by incubating various liposome types with DPCs for 2 and 6 h (Fig. 4C). Both LP and L-LP exhibited enhanced fluorescence signals with increased incubation. Notably, the fluorescence intensity of the L-LP group surpassed that of the LP group at all time points, corroborating the FCM results (Fig. 4C). Compared with that of the control group, the average fluorescence intensity of the cells treated with L-LPs increased by 8.4% and 13.4% after 2 and 6 h, respectively. These findings indicate that leptin modification enhances liposome–DPC interactions and promotes cellular internalization.

To further explore the targeting mechanism, LEPR was labeled with Alexa Fluor 488 (green), while L-LPs were visualized by red fluorescence. Colocalization of LEPR and L-LP signals within HFs was observed (Fig. 4D), suggesting that leptin-mediated recognition facilitates localized accumulation of liposomes in proximity to DPCs. Together, these results indicate that L-LP leverages both carrier-mediated permeability and receptor-associated targeting to enhance follicular localization and retention at the intended therapeutic site.

### In vitro effect of L-LP-Fi/CeNPs on DPC senescence

AGA is primarily characterized by the conversion of T to DHT, which subsequently leads to the senescence and dysfunction of DPCs. Initially, we determined the cytotoxicity of finasteride and CeNPs on DPCs, as well as potential drug–drug interactions. The result from CCK-8 assays indicated that both drugs, whether administered individually or in combination, exhibited noncytotoxic properties at specific concentrations. Meanwhile, we systematically evaluated the combined effect of finasteride and CeNPs by constructing a dose–response matrix and applying 3 computational models, namely, Bliss, highest single agent, and Loewe for cross-validation. All models consistently demonstrated that co-treatment at a 1:1 ratio produced a statistically synergistic effect (Fig. S3A and B). Isobolographic analysis further confirmed that data points within key dose combinations deviated from the additive effect reference line and consistently fell within the synergy region (Fig. S3C). Together, these multimodel validation results provide coherent evidence that the drug combination produces a substantive synergistic effect with statistical significance, thereby supporting the hypothesis that finasteride and CeNPs act synergistically to mitigate T-induced DPC mortality.

**Fig. 5.**
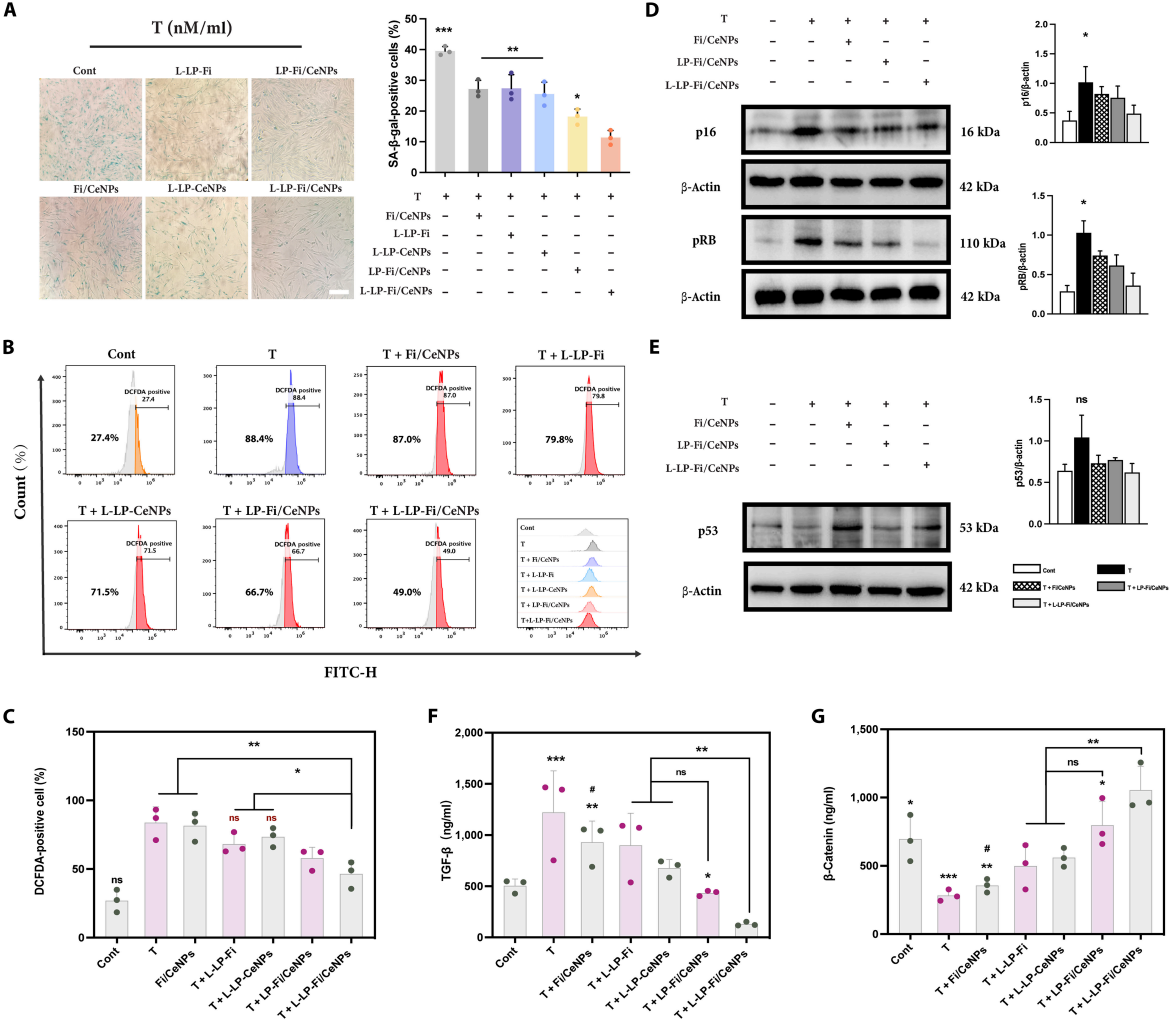
Effect of L-LP-Fi/CeNPs on DPC senescence. (A) HDPCs were treated with different drugs for 12 h and incubated with T for 12 h. Senescent cells were evaluated for SA-β-gal activity. Positive cells (blue) in 5 randomized regions were counted manually. The proportion of positive cells is expressed as a percentage of cells in each treatment group (*n* = 5). Scale bar = 150 μm. (B) HDPCs were subjected to differential treatment for 6 h and then treated with T for 2 h. The cells were subsequently stained with 2′,7′-dichlorodihydrofluorescein diacetate (H2DCFDA) (1 μM) and analyzed via FCM. (C) Quantification of the number of ROS-positive cells in (B) (*n* = 3). (D and E) Cells were pretreated with different drugs for 12 h and then treated with T for 12 h. The protein expression levels of p16, pRb, and p53 were analyzed by Western blot. β-Actin was used as a loading control (*n* = 3). (F) TGF-β1 and (G) β-catenin expression after pretreatment with different drugs for 12 h and then treatment with T for 12 h (*n* = 3). ns, nonsignificant (*P* > 0.05); *P < 0.05, **P < 0.01, and ***P < 0.001 vs L-LP-Fi/CeNPs. ^##^*P* < 0.01 vs LP-Fi/CeNPs. SA-β-gal, senescence-associated β-galactosidase; FITC, fluorescein isothiocyanate; DCFDA, 2′,7′-dichlorofluorescein diacetate.

Subsequently, we conducted a SA-β-gal activity assay to evaluate the senescence state of the cells. The results indicated that drug treatment resulted in a reduction in the proportion of SA-β-gal-positive DPCs, with the lowest percentage recorded in cells pretreated with L-LP-Fi/CeNPs (Fig. 5A). Given that elevated levels of ROS are indicative of senescent cells, we analyzed the ROS levels in T-treated DPCs using FCM. Our data revealed a decrease in ROS levels following pretreatment with various drugs, with L-LP-Fi/CeNPs achieving the most substantial reduction in ROS accumulation, quantified at 38.1% (Fig. 5B and C).

Moreover, pretreatment with L-LP-Fi/CeNPs markedly suppressed the expression of the senescence markers p16 and pRb in T-treated DPCs (Fig. 5D). Notably, Western blot analysis indicated that L-LP-Fi/CeNPs ameliorated T-induced DPC senescence primarily via the p16/pRb signaling pathway, rather than the p53 pathway (Fig. 5E). From a mechanistic perspective, cellular senescence in DPCs in the context of AGA is widely regarded as being mainly associated with activation of the p16INK4a/pR pathway, rather than the canonical p53-dependent DNA damage response [9,39]. In this study, the marked suppression of the p16/pRb pathway without statistically notable changes in the p53 pathway suggests that our intervention specifically reverses the p16-mediated senescence program, which is most relevant to the pathogenesis. Given that DPC senescence alters the secretion of growth factors critical for the proliferation and activation of HFSCs, we measured the levels of TGF-β1 (a cytokine that induces apoptosis and inhibits HFSC activation; see Fig. 5F) and β-catenin (a cytokine that promotes cell proliferation and HFSC activation; see Fig. 5G) following T treatment. The results demonstrated that pretreatment with L-LP-Fi/CeNPs effectively reversed the increase in the TGF-β1 levels and the decrease in the β-catenin levels. These findings confirm that L-LP-Fi/CeNPs markedly alleviate T-induced senescence in DPCs and modulate the secretion of factors associated with the HF cycle.

### Evaluation of hair growth induced by L-LP-Fi/CeNPs in an AGA model

**Fig. 6.**
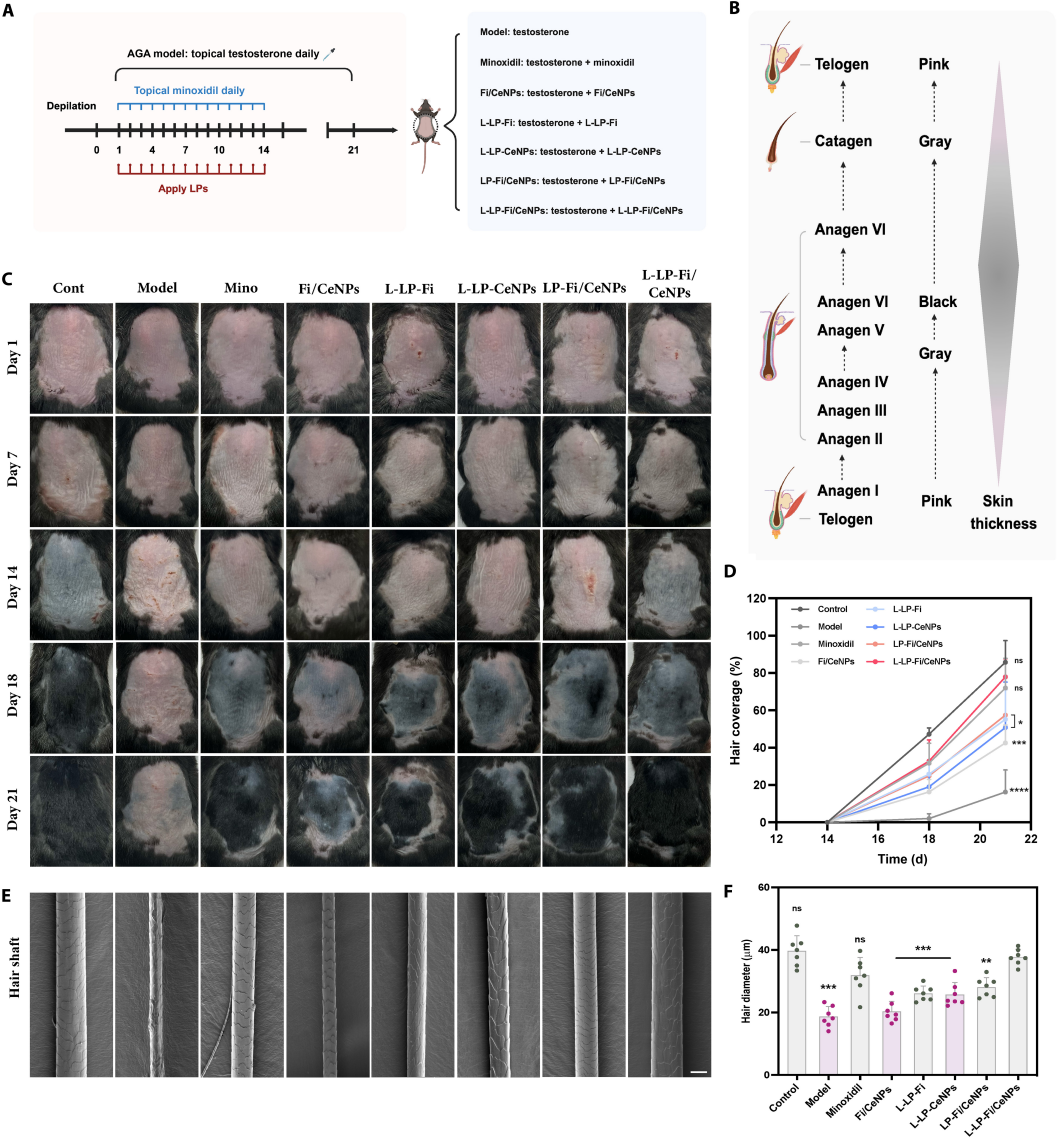
Hair regeneration efficiency of the L-LP-Fi/CeNP system in AGA mice. (A) Schematic representation of the treatment protocol. An AGA model was established by topically applying a 0.5% testosterone solution for 21 consecutive days. The control group did not receive any drugs postdepilation. The other groups were administered a testosterone solution daily, followed by the appropriate therapeutic drug a few hours later. (B) Time scale of the hair cycle in C57BL/6 mice and changes in skin pigmentation and skin thickness after depilation. (C) Representative photographs of the depilated regions on days 1, 7, 14, and 21 postdepilation in each treatment group. (D) Hair coverage rate after epilation (n = 4). (E) SEM images of the regenerated hair on day 21 postdepilation. Scale bar = 50 μm. (F) Diameter of regenerated hair at day 21 postdepilation (n = 8). All results are presented as the means ± SDs. ns, nonsignificant (*P* > 0.05), *P < 0.05, **P < 0.01, and ***P < 0.001 versus L-LP-Fi/CeNPs. ^##^P < 0.01 versus LP-Fi/CeNPs. Mino, minoxidil.

To evaluate the hair-growth-promoting effects of the L-LP-Fi/CeNP nanomodulator in vivo, an AGA model was established, with treatment groups assigned as shown in Fig. 6A. Photograph documentation of hair growth was conducted on days 1, 7, 14, 18, and 21. Hair regrowth was observed in the model group until day 21 (Fig. 6A), confirming the successful establishment of the AGA model.

To address the potential bioactivity of the leptin ligand, we introduced an L-LP-only control group. This group showed no significant therapeutic advantage over the model group in terms of anagen initiation or final hair coverage (Fig. S4), indicating that the observed efficacy is primarily mediated by the drug cargo (Fi and CeNPs) rather than leptin–LEPR signaling-mediated effects.

As the hair cycle transitions from the telogen phase to the anagen phase, skin pigmentation occurs due to melanogenesis within HFs (Fig. 6B), serving as a visual indicator of the cycle transition [40,41]. Except for the control group, the L-LP-Fi/CeNP group exhibited the most rapid entry into the anagen phase by day 14 compared with the other treatment groups (Fig. 6C and Fig. S5). By day 21, the hair coverage achieved with L-LP-Fi/CeNP treatment (78.6%) was comparable to, and slightly higher than, that achieved with minoxidil (72.0%) while remaining lower than that of the normal control group (85.8%). Furthermore, the performance of the LP-Fi/CeNP group (57.5%) surpassed that of the free drug group (40.0%), highlighting the efficacy of liposomes as drug delivery carriers. A comparative analysis between the L-LP-Fi/CeNP group and the L-LP-Fi (55.19%) and L-LP-CeNP (50.0%) groups illustrated the benefits of combined drug therapy. The observed disparity in hair coverage between the L-LP-Fi/CeNP and LP-Fi/CeNP groups further emphasized the effectiveness of this targeted delivery system in enhancing hair growth promotion (Fig. 6D).

SEM analysis revealed that the diameter of neonatal hairs in the L-LP-Fi/CeNP group was most closely aligned with that of the control group (Fig. 6E). Additionally, the regenerated hair cuticles in the L-LP-Fi/CeNP group were intact and exhibited a shiny appearance, in contrast to the dry and unhealthy appearance of hair cuticles in the model and free drug groups (Fig. 6F).

In addition to efficacy, a comprehensive safety assessment was conducted to investigate potential immune responses triggered by repeated topical application. Histopathological examination of major organs (heart, liver, spleen, lungs, and kidneys) revealed no observable abnormalities or treatment-related lesions in any group (Fig. S6A), indicating the absence of overt systemic toxicity. To obtain an initial assessment of potential systemic effects, including immune activation—a key concern for nanoformulations—we conducted a complete blood count analysis. The results demonstrated that all key hematological parameters, including the white blood cell count, remained within normal ranges and showed no significant differences between the L-LP-Fi/CeNP group and the normal control group (Table S3). Together, these results support the short-term systemic biocompatibility of the L-LP-Fi/CeNP formulation under the experimental conditions used in this study.

### In vivo assessment of L-LP-Fi/CeNPs in HF cycle regulation

**Fig. 7.**
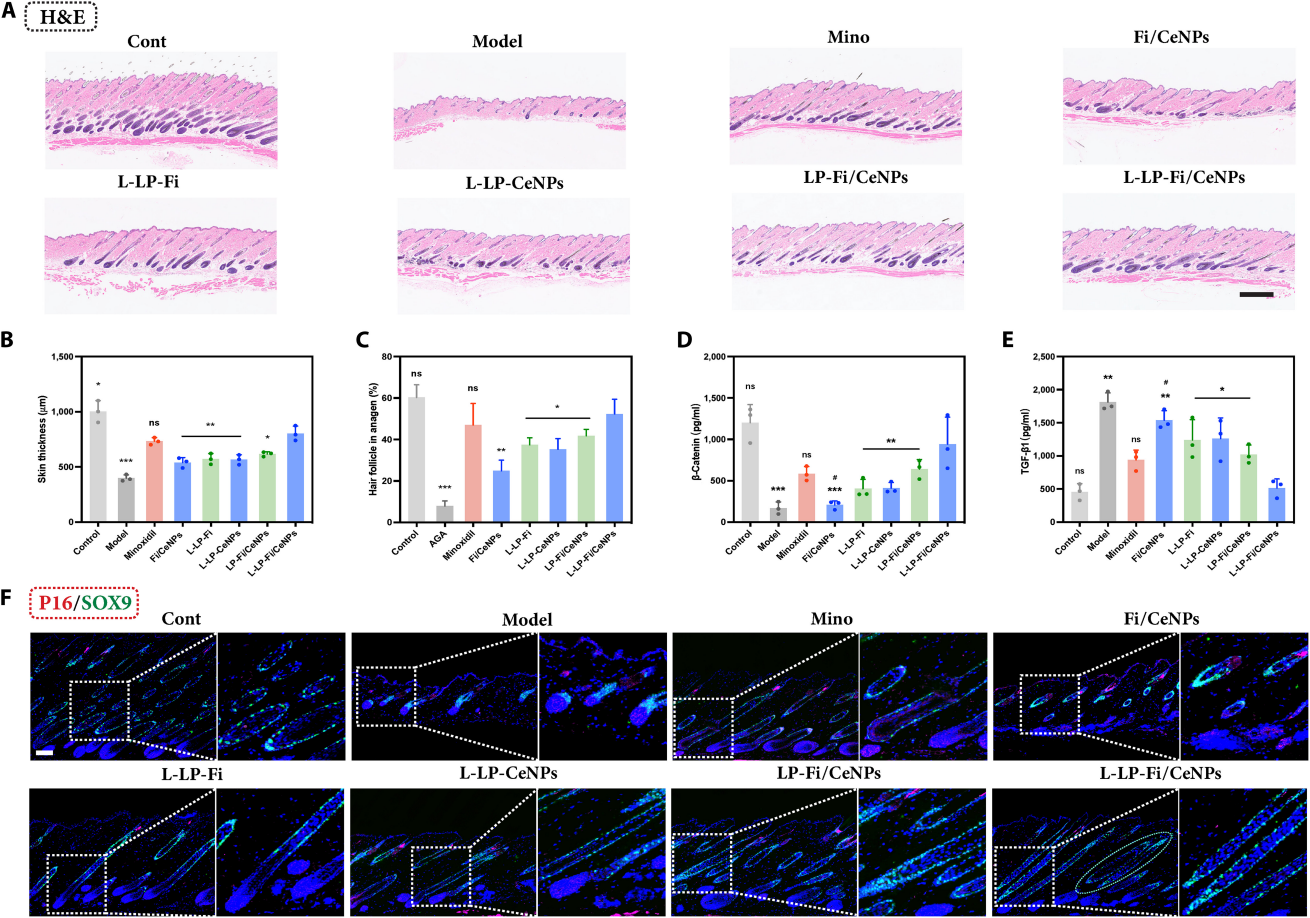
HF cycle regulation. (A) H&E staining of the treated skin on day 14 postdepilation. Scale bar = 500 μm. (B) Skin thickness on day 14 postdepilation (*n* = 3). (C) Quantification of HFs in the anagen phase (*n* = 10). (D) β-Catenin and (E) TGF-β1 expression in the skin on day 14 postdepilation (*n* = 3). (F) Representative images of immunofluorescence staining for SRY-box transcription factor 9 (SOX9) and p16 in treated skin on day 14 postdepilation (green, SOX9; red, p16; and blue, DAPI). Scale bar = 200 μm. All results are presented as means ± SDs. ns, nonsignificant (*P* > 0.05). *P < 0.05, **P < 0.01, and ***P < 0.001 vs L-LP-Fi/CeNPs. H&E, hematoxylin and eosin.

H&E staining serves as a valuable method for assessing the status of and changes in the HF cycle, as evidenced by changes in skin thickness and HF morphology. On day 14, H&E staining (Fig. 7A) revealed that HFs in the control group exhibited notable growth in hair bulb size due to the rapid division and proliferation of dermal matrix cells and DPCs during the anagen phase, leading to increased skin thickness. Moreover, the skin thickness (Fig. 7B) in the L-LP-Fi/CeNP group was greater than those in the other treatment groups and was comparable to that of the minoxidil group. Morphological analysis indicated that the HFs in the control group were elongated, straight, and uniformly oriented, indicating a distinct anagen state. In contrast, the HFs in the modeling group presented characteristic changes associated with AGA, manifested as miniaturized bulbs in the superficial skin layers, indicating a telogen or an early anagen phase. Notably, except for those in the control group, the HFs in the L-LP-Fi/CeNP group extended into the subcutaneous tissue with greater volume and density, signifying a rapid progression into the anagen phase. The HFs in the L-LP-Fi/CeNP group predominantly exhibited anagen phases IIIb, IIIc, and V, which were intermediate between those observed in the control group (anagen phases V and VI) and the minoxidil group (anagen phases IIIa, IIIb, and IIIc). This observation aligns with the hair cycle scores (Fig. 7C).

The Wnt/β-catenin and bone morphogenetic protein/TGF-β signaling pathways play crucial roles in regulating the phases of the HF cycle. To further elucidate the dynamics of the HF growth cycle, we investigated the levels of β-catenin and TGF-β1 in the dorsal skin of mice on day 14. The relative expression level of β-catenin (Fig. 7D) was considerably increased in the L-LP-Fi/CeNP group. In contrast, the expression level of TGF-β1 (Fig. 7E) was lower than that in the model group. These findings provide compelling evidence that L-LP-Fi/CeNPs facilitate the transition of the HF cycle.

Additionally, p16 (a marker of cellular senescence) and SOX9 (a marker of HFSC activation) were evaluated via fluorescence labeling to assess the progression of the HF cycle (Fig. 7F). In both the control and L-LP-Fi/CeNP groups, SOX9 exhibited a circular distribution surrounding the HFs, indicating the activation of HFSCs and their migration toward the lower HFs to initiate the anagen phase, while p16 expression remained confined to the superficial skin layer. In contrast, the model group displayed pronounced SOX9 fluorescence in the upper bulge region, suggesting that HFSCs remained in a quiescent state, whereas p16 expression markedly increased in DPCs. These findings indicate that L-LP-Fi/CeNPs effectively promote HFSC activation, inhibit DPC senescence, and facilitate the coordinated progression of the HF cycle.

### In vivo evaluation of the efficacy of L-LP-Fi/CeNPs in modifying the perifollicular microenvironment

**Fig. 8.**
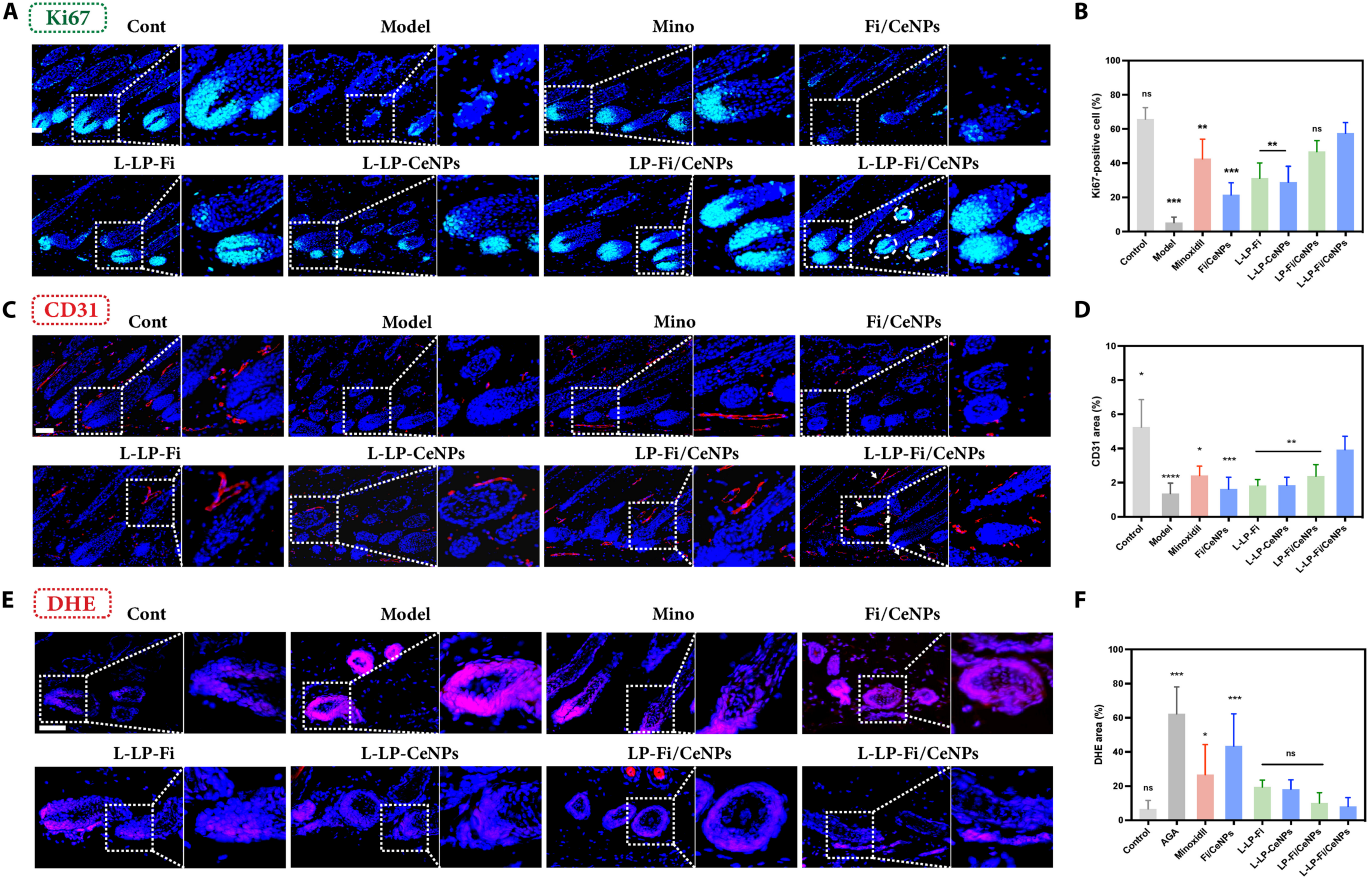
Evaluation of the ability of L-LP-Fi/CeNPs to reshape the perifollicular microenvironment. (A) Representative images of Ki67 in skin tissues from different groups on day 14 postdepilation (green: Ki67; blue: DAPI). Scale bar = 100 μm. (B) Ki67 expression in HFs (*n* = 10 viewing fields). (C) Representative images of CD31 in skin tissues from different groups on day 14 postdepilation (red: CD31; blue: DAPI). Scale bar = 100 μm. (D) CD31 expression in the microenvironment (*n* = 10 viewing fields). (E) Representative images of DHE in skin tissues from different groups on day 14 postdepilation were used to detect ROS. Scale bar = 20 μm. (F) Quantitative analysis of oxidized DHE on day 14 postdepilation (*n* = 10 viewing fields). All results are presented as means ± SDs. ns, nonsignificant (*P* > 0.05). *P < 0.05, **P < 0.01, and ***P < 0.001 vs L-LP-Fi/CeNPs. DHE, dihydroethidium.

In AGA, the perifollicular microenvironment undergoes profound alterations, including disruptions in cellular signaling pathways, alterations in growth factor secretion, reduced angiogenesis, and impaired DPC functionality, all of which adversely affect the HF cycle. This study aimed to assess the impact of the L-LP-Fi/CeNPs’ precision dual drug delivery system on the remodeling of the perifollicular microenvironment. Initial analyses of molecular markers related to perifollicular vascularization and nutrient supply substantiated the role of L-LP-Fi/CeNPs in this remodeling process. Notably, the expression of Ki67, a marker indicative of cellular proliferation, was markedly elevated in the dermal papilla region treated with L-LP-Fi/CeNPs (Fig. 8A and B), indicating an enhanced environment conducive to DPC proliferation. Additionally, the expression of CD31, a marker for vascular proliferation, exhibited a significant increase following treatment with L-LP-Fi/CeNPs (Fig. 8C and D). The formation of a ring-shaped vascular network surrounding the HFs indicates stimulated angiogenesis and the generation of new vascular endothelial cells, thereby supporting the progression of the HF cycle.

Considering the in vitro ROS-scavenging capabilities of CeNPs, we assessed the intracellular oxidative stress levels using DHE staining (Fig. 8E). The model group displayed pronounced DHE fluorescence, indicative of a heightened oxidative stress within the HF microenvironment under AGA conditions. Conversely, the minoxidil-treated group demonstrated a reduced DHE fluorescence, likely due to its superoxide-scavenging properties and inhibitory effects on lipid peroxidation. Importantly, the L-LP-Fi/CeNP group demonstrated the most substantial reduction in intracellular ROS levels, indicating that CeNPs effectively replicate the actions of SOD and CAT in vivo (Fig. 8F).

Collectively, these results indicate that L-LP-Fi/CeNPs modulate key components of the perifollicular microenvironment, including cellular proliferation, angiogenesis, and oxidative stress, which are closely associated with HF cycling and regeneration.

## Discussion

AGA is a disorder of scalp hair growth characterized by progressive miniaturization of HFs and a reduction in the number of actively growing follicles [42]. The pathogenesis of AGA is complex and involves multiple contributing factors. Recent studies have established that DPCs play a central role in driving the hair cycle transition by maintaining a precise balance between activating and inhibitory signals. Specifically, the Wnt/β-catenin pathway acts as a key molecular switch for initiating the anagen phase, promoting DPCs to secrete growth factors such as vascular endothelial growth factor (VEGF) and fibroblast growth factor 7 (FGF7), which in turn activate HFSCs to launch a new regenerative cycle [43]. In contrast, bone morphogenetic protein signaling maintains telogen homeostasis by suppressing the activation of HFSCs, and fibroblast growth factor 18 (FGF-18) has been shown to prolong the quiescent state of follicles [44]. The spatiotemporally precise regulation of these signaling pathways collectively ensures the normal rhythm of the hair cycle [45].

Emerging evidence has revealed that premature senescence of DPCs is a critical mechanism underlying the disruption of follicular regulation. Under multiple stressors, including sustained stimulation by DHT, oxidative stress, and overexpression of the AR, DPCs undergo irreversible cell cycle arrest via key pathways such as p16/pRb, accompanied by a remodeling of the senescence-associated secretory phenotype [46]. This senescence-associated secretory phenotype is characterized by up-regulated expression of inhibitory factors such as TGF-β and interleukin-6 (IL-6), along with down-regulated expression of growth-promoting factors like β-catenin, ultimately disrupting the microenvironmental homeostasis necessary for HF regeneration [47,48]. Therefore, targeting DPCs to inhibit their senescence and restore their physiological function may represent a potential therapeutic strategy for intervening in follicular miniaturization and promoting hair regeneration.

Single-cell transcriptomic profiling, corroborated by our preliminary experimental data, revealed that LEPR is specifically and highly expressed in DPCs, with this expression pattern remaining stable throughout the distinct phases of the HF cycle [32,49]. This finding suggested LEPR as a potential molecular anchor for follicle-targeted delivery. To experimentally validate its targeting feasibility, we performed immunofluorescence staining coupled with confocal microscopy, which confirmed the enriched distribution of LEPR on the DPC and its efficient binding capability with a targeting ligand. Our results demonstrate that LEPR possesses not only the specificity required for DPC identification but also the functional capacity to mediate ligand–receptor binding, thereby enabling precise drug delivery. Consequently, LEPR can be regarded as a suitable molecular target for DPC-focused delivery, providing a translational opportunity to leverage its biological specificity for therapeutic targeting.

Based on these considerations, we constructed a functionalized liposomal system co-encapsulating finasteride and cerium oxide nanozymes to enable targeted intervention at the level of DPC senescence. This strategy integrates LEPR-mediated targeting with coordinated modulation of androgen signaling and oxidative stress, 2 key drivers of premature DPC aging. Unlike conventional therapies that primarily act on downstream manifestations, this approach intervenes upstream at the p16/pRb-associated senescence axis, thereby partially restoring the hair-inductive capacity of DPCs and supporting the activation of HFSCs. Co-loading finasteride and cerium oxide nanozymes into a follicle-retentive liposomal carrier further enables localized and relatively sustained therapeutic action, reflecting the cell-specific and mechanism-oriented design of this strategy. In this context, the approach does not rely on innovation in any single therapeutic component; rather, it adopts a disease-mechanism-driven integrative design that organizes established elements into a senescence-centered intervention pathway directed toward functional restoration of DPCs.

Nevertheless, several considerations remain relevant for clinical translation. AGA is a chronic condition requiring long-term and repeated intervention, yet most nanomaterial-based studies, including the present work, primarily emphasize short-term efficacy and preliminary safety. Accordingly, the long-term local tolerability and toxicological behavior of follicle-targeted nanotherapeutic systems warrant further investigation under extended dosing conditions. In addition, although liposomes with mean diameters of approximately 150 to 200 nm were employed based on commonly used ranges in follicular delivery studies, systematic size-optimization comparisons were not performed and may further inform translational refinement. Finally, interspecies differences in hair cycle dynamics and skin architecture, together with patient adherence considerations, remain important factors when extrapolating preclinical findings to clinical application.

## Data Availability

Data will be made available upon request.
