# Supplementary material for: Oxidative Stress and Hormone-Regulated Dermal Papilla Cell-Targeted Nanomodulators: Reverse Cellular Senescence for Androgenetic Alopecia Therapy
Source: Biomater Res. 2026 Mar 11;30:0333. doi: 10.34133/bmr.0333 (PMC12976380; doi:10.34133/bmr.0333)
Supplement: Supplementary 1 — Figs. S1 to S6 Tables S1 to S3 [file bmr.0333.f1.docx]

*Supporting Information*

**Oxidative Stress and Hormone-regulated Dermal Papilla Cell-targeted Nanomodulators: Reverse Cellular Senescence for Androgenetic Alopecia Therapy**

*Lan Lan^1, †^, Qingde Zhou^1, 2, †^, Shuangxue Pan^3^, Hui Liu^4^, Yongzhong Du^5^, Cuiping Guan^4, *^, Xiuzu Song ^1, 4, *^, Wei Wang ^2,1, 4, *^*

^1^ Department of Dermatology, Affiliated Hangzhou Dermatology Hospital, Zhejiang University School of Medicine, Hangzhou Third People’s Hospital, Hangzhou 310009, China.

^2^ Department of Pharmacy, Affiliated Hangzhou Dermatology Hospital, Zhejiang University School of Medicine, Hangzhou Third People’s Hospital, Hangzhou 310009, China.

^3^ School of Pharmacy, Hangzhou Normal University, Hangzhou, Zhejiang 311121, China

^4^ Hangzhou Third Hospital Affiliated Zhejiang Chinese Medical University, Hangzhou, 310009, China

^5^ State Key Laboratory of Advanced Drug Delivery and Release Systems, Institute of Pharmaceutics, College of Pharmaceutical Sciences, Zhejiang University, Hangzhou 310058, China.

*Corresponding author(s).

Tel: +86 13777476467

E-mail addresses: [wangzi0209@zju.edu.cn](mailto:wangzi0209@zju.edu.cn) (Wei Wang), [songxiuzu@sina.com](mailto:songxiuzu@sina.com) (Xiuzu Song), imgcp@zcmu.edu.cn (Cuiping Guan).

^†^ These authors contributed equally.

**This file includes:**

Supporting Figures S1 to S6

Supporting Tables S1 and S3

**Supplementary Information**

**
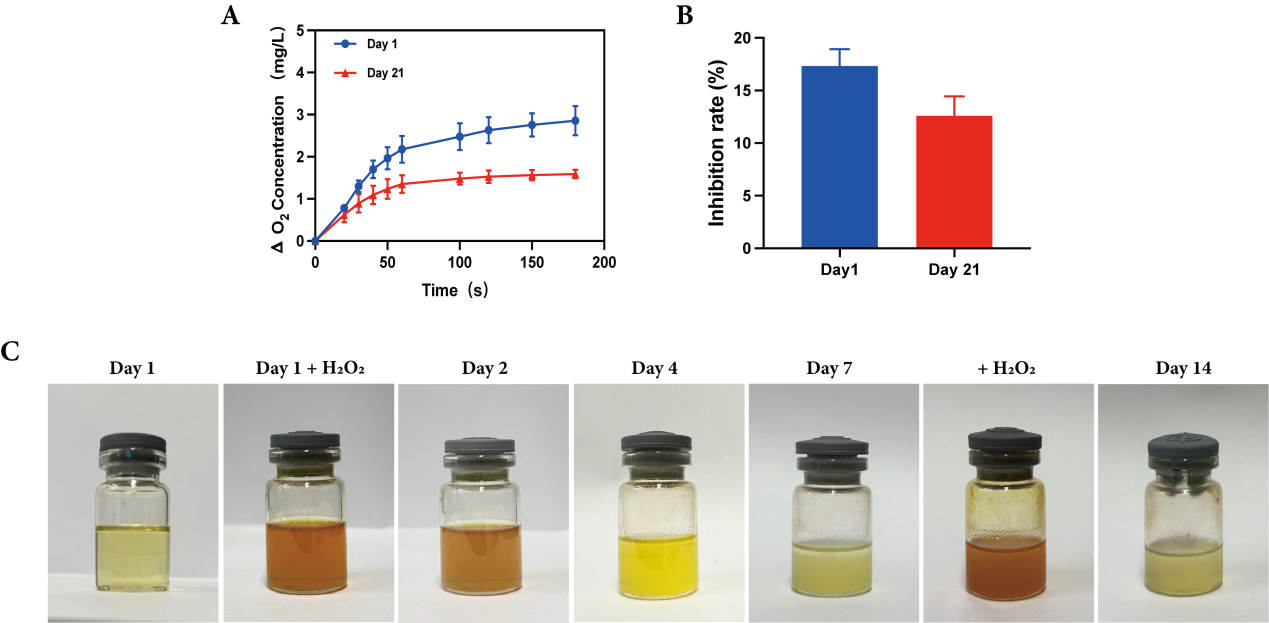
**

**Figure S1.** (A) CAT and (B) SOD activities at day 21. (C) The valence cycling ability of CeNPs.

**
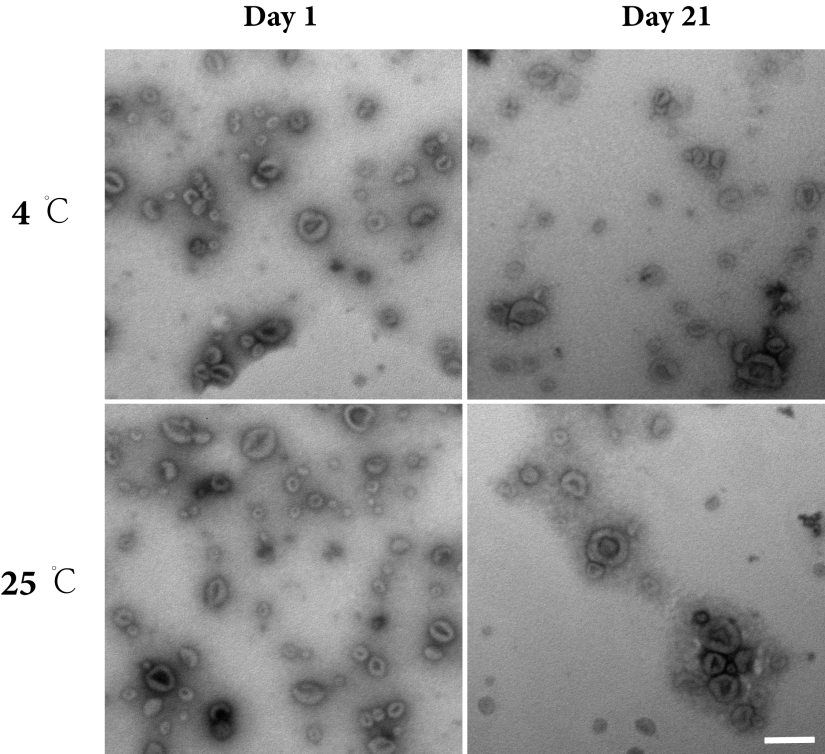
**

**Figure S2.** Long-Term Morphological Stability.Scale bar = 100 µm.


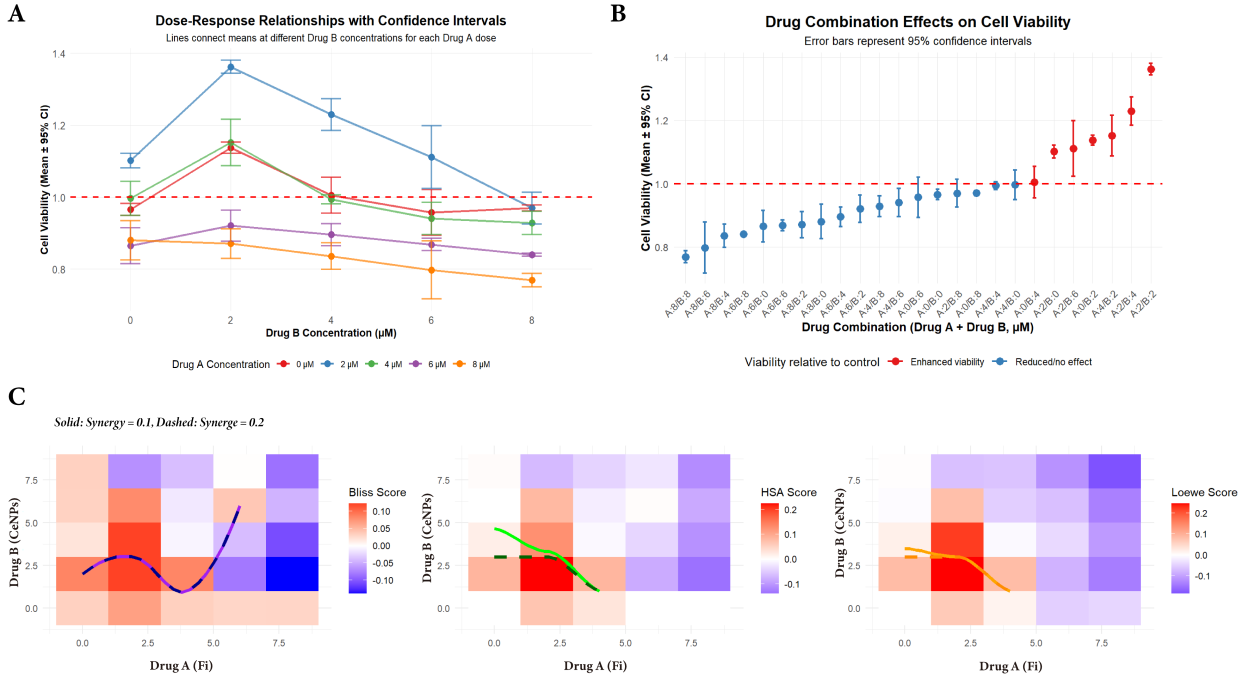


**Figure S3.** Analysis of Drug Synergy between Finasteride and CeNPs using the Bliss Independence, HSA, and Loewe Models. (A) Dose-dependent effects of Finasteride and CeNPs on cell viability. (B) Combined treatment profiles showing synergistic interactions between Finasteride and CeNPs across concentration gradients. (C) Heatmaps of synergy scores generated using the Bliss, HSA, and Loewe models.


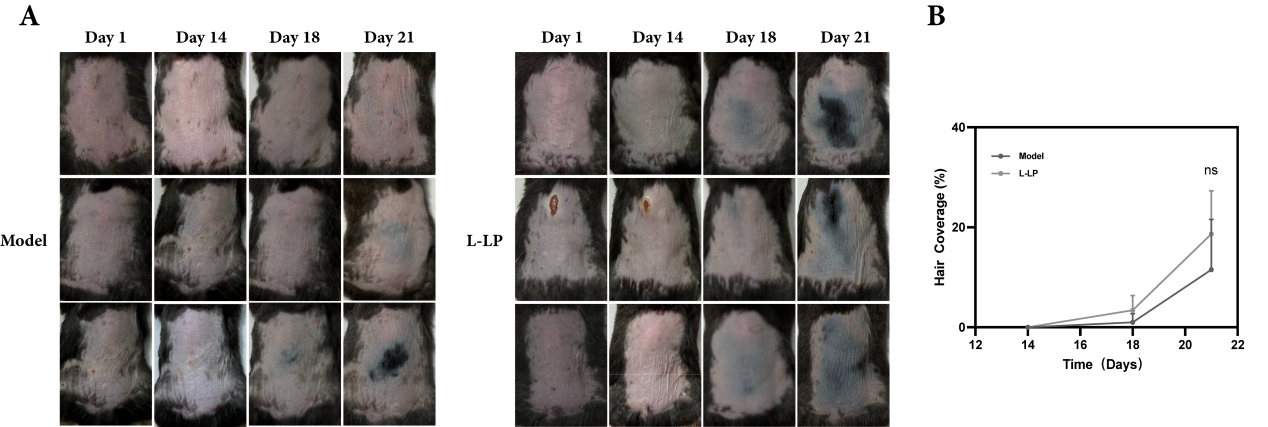


**Figure S4.** *In Vivo* Hair Growth Assessment of Leptin-Functionalized Liposomes (L-LP). n=3 .


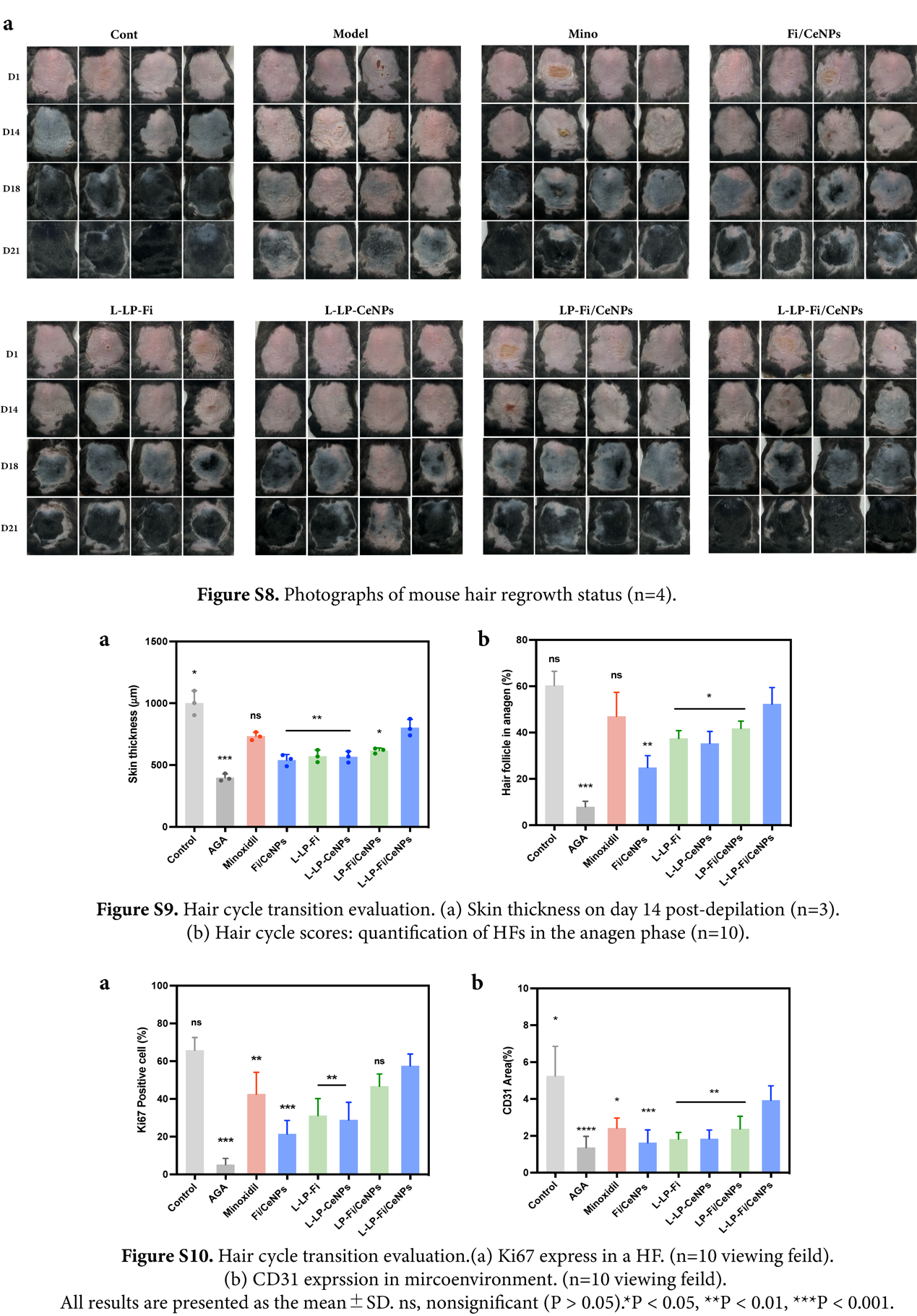


**Figure S5.** Photographs of mouse hair regrowth status (n = 4).

**Figure S6.** Long-Term Safety Evaluation of L-LP-Fi/CeNPs. Histological analysis of major organs (heart, lung, liver, kidney,and spleen) from AGA model mice after 21 days of treatment with PBS, T, L-LP, and L-LP-Fi/CeNPs. Organ sections were processed for H&E staining. Scale bar = 200 µm.

**Table S1.** Table of Abbreviations

| Abbreviation | Full English Term |
| --- | --- |
| AGA | Androgenetic alopecia |
| AR | Androgen receptor |
| CCK-8 | Cell counting kit-8 |
| CD31 | Platelet endothelial cell adhesion molecule-1 |
| CLSM | Confocal laser scanning microscope |
| DAPI | 4’,6-diamidino-2-phenylindole |
| DHE | Dihydroethidium |
| DHT | Dihydrotestosterone |
| DiI | 1,1’-dioctadecyl-3,3,3’,3’-tetramethylindocarbocyanine perchlorate |
| DPCs | Dermal papilla cells |
| DSPE-PEG-NHS | 1,2-Distearoyl-sn-glycero-3-phosphoethanolamine-polyethylene glycol-polyethylene glycol-succinimide ester |
| ELISA | Enzyme-linked immunosorbent assay |
| Fi | Finasteride |
| FITC | Fluorescein Isothiocyanate |
| HaCaT | Human-derived keratinocytes |
| HDPC | Human-derived dermal papilla cell |
| HPLC | High performance liquid chromatography |
| Ki67 | Proliferation related Ki-67 antigen |
| LEPR | Leptin receptor |
| MDPC | Mouse-derived dermal papilla cell |
| mEK | Murine-derived keratinocytes |
| SA-β-gal | Senescence-Associated β-Galactosidase |
| SOX9 | Campomelic dysplasia autosomal sex reversal |
| TGF-β1 | Transforming growth factor-β1 |
| FGF-18 | Fibroblast Growth Factor-18 |

**Table S2.** 21-day stability analysis of L-LP-Fi/CeNPs.

| **Storage temperature** | **Parameter** | 1day | 7day | 21day |
| --- | --- | --- | --- | --- |
| 4 ℃ | DLS (nm) | 177.5 | 179.3 | 185.9 |
|  | PDI | 0.22 | 0.24 | 0.36 |
|  | EE (%) | 67.7 | 65.9 | 60.9 |
| 25 ℃ | DLS (nm) | 163.7 | 172.2 | 182.9 |
|  | PDI | 0.22 | 0.27 | 0.41 |
|  | EE(%) | 64.1 | 60.3 | 56.6 |

**Table S3.** Hematological analysis of L-LP and L-LP-Fi/CeNPs.

| Hematological indexes | Control | Model | L-LP | L-LP-Fi/CeNPs |
| --- | --- | --- | --- | --- |
| RBC (10^12/L) | 8.5 ± 0.3 | 8.4 ± 0.8 | **8.2 ± 0.2** | **8.2 ± 0.2** |
| HGB (g/L) | 146 ± 3 | 147 ± 3 | **148 ± 3** | **146 ± 3** |
| MCV (fL) | 41.9 ± 0.7 | 41.7 ± 0.9 | **42.0 ± 0.5** | **42.1 ± 0.4** |
| HCT (%) | 34.7 ± 1.0 | 34.4 ± 0.8 | **34.5 ± 0.8** | **34.3 ± 0.6** |
| MCH (pg) | 15.7 ± 0.1 | 16.4 ± 0.2 | **16.9 ± 0.2** | **16.9 ± 0.2** |
| MCHC (g/L) | 422 ± 7 | 415 ± 10 | **427 ± 4** | **429 ± 2** |
| WBC (10^9/L) | 8.4 ± 0.9 | 7.1 ± 1.0 | **7.2 ± 0.9** | **7.3 ± 0.8** |
| PLT (10^9/L) | 418 ± 63 | 428 ± 55 | **426 ± 45** | **422 ± 40** |
